# Retrospective study of comparable survival after neoadjuvant versus adjuvant chemotherapy in cT1-2N0M0 triple-negative breast cancer

**DOI:** 10.1007/s10549-026-07983-9

**Published:** 2026-05-12

**Authors:** Maxim Olsson, Slavica Janeva, Jari Martikainen, Anna-Karin Tzikas, Per Karlsson, Toshima Z. Parris

**Affiliations:** 1 Department of Oncology, Institute of Clinical Sciences, Sahlgrenska Academy, University of Gothenburg, Gothenburg, Sweden; 2 Sahlgrenska Center for Cancer Research, Sahlgrenska Academy, University of Gothenburg, Gothenburg, Sweden; 3 Region Västra Götaland, Department of Surgery, Sahlgrenska University Hospital, Gothenburg, Sweden; 4 Department of Surgery, Institute of Clinical Sciences, Sahlgrenska Academy, University of Gothenburg, Gothenburg, Sweden; 5 Bioinformatics and Data Centre, Sahlgrenska Academy, University of Gothenburg, Gothenburg, Sweden; 6 Region Västra Götaland, Department of Oncology, NU Hospital Group, Uddevalla, Sweden; 7 Region Västra Götaland, Department of Oncology, Sahlgrenska University Hospital, Gothenburg, Sweden

**Keywords:** Survival analysis, Charlson comorbidity index, Systemic therapy, Breast-conserving surgery, Real-world data, Epidemiology

## Abstract

**Purpose:**

Triple-negative breast cancer (TNBC) is an aggressive subtype commonly treated with chemotherapy and radiotherapy, administered preoperatively as neoadjuvant chemotherapy (NACT) or postoperatively as adjuvant treatment (AT; defined here as adjuvant chemotherapy [ACT] with or without adjuvant radiotherapy [ART]). As NACT is increasingly favored, the relative survival outcomes of these approaches and the added benefit of postoperative therapy after NACT remain uncertain. This study aimed to assess their impact on survival.

**Methods:**

In this nationwide registry-based cohort study, data for women diagnosed with cT1–2N0M0 TNBC in Sweden between 2007 and 2021 were retrieved from the Swedish National Quality Register for Breast Cancer. Survival outcomes for patients receiving NACT ± AT were compared with those receiving AT only. Propensity score matching (1:1) was performed, adjusting for age, clinical T-stage, and comorbidity. Overall survival (OS) and breast cancer-specific survival (BCSS) were estimated using Kaplan–Meier and Cox proportional hazards models.

**Results:**

Of 3747 eligible patients, 711 received NACT ± AT and 3036 received AT alone. Median follow-up for BCSS was 3.61 years (IQR 2.37–5.50) for NACT and 6.95 years (IQR 4.36–9.62) for AT. After matching, 711 patients remained in each group. Both prior and post matching, 5-year OS and BCSS did not differ significantly between AT and NACT. These findings remained consistent after adjustment for potential confounders.

**Conclusion:**

OS and BCSS were similar between AT and NACT. These findings suggest that chemotherapy sequencing was not associated with a detectable survival difference, although treatment selection should be individualized and evaluated in the context of contemporary regimens.

**Supplementary Information:**

The online version contains supplementary material available at 10.1007/s10549-026-07983-9.

## Introduction

Triple-negative breast cancer (TNBC) is characterized by the absence of estrogen and progesterone receptors (ER and PgR), the lack of overexpression or gene amplification of human epidermal growth factor receptor 2 (HER2), making TNBC the only major breast cancer subtype without targeted therapies [1]. TNBC is overrepresented among premenopausal women and certain ethnic groups, particularly Black and Hispanic populations, and accounts for about 10% of breast cancers, depending on national thresholds for ER and PgR, and current guidelines for HER2 subclassification into HER2-null, HER2-ultralow, and HER2-low [2–4]. Despite advances in multimodal therapy, surgery together with chemotherapy, with or without radiotherapy, remains the standard treatment for early-stage TNBC. Nevertheless, around 30% of patients still experience distant relapse within the first 3 years, whereas in other breast cancer subtypes relapses more often occur later, highlighting ongoing therapeutic challenges [5, 6].

During the 1990s, the National Surgical Adjuvant Breast and Bowel Project (NSABP; studies B-18 and B-27) investigated the potential benefits of preoperative neoadjuvant chemotherapy (NACT) versus postoperative adjuvant chemotherapy (ACT) that was already used in routine practice [7–9]. In 2014, the CTNeoBC pooled analysis of NACT-related clinical trials in breast cancer revealed that pathological complete response (pCR) correlates with improved survival in patients with TNBC and HER2-positive disease [10]. Moreover, NACT facilitates the assessment of chemosensitivity, with pCR being a surrogate marker of long-term survival, especially in TNBC and HER2-positive disease. Initially reserved for inoperable and locally advanced breast cancer, NACT is now increasingly used to downstage operable cases, enable breast-conserving surgery (BCS), achieve pCR, reduce surgical morbidity, and target micrometastases [11]. Moreover, a meta-analysis by the Early Breast Cancer Trialists’ Collaborative Group showed that NACT was associated with a higher rate of BCS (65%) than ACT regimen (49%) [12].

According to the Swedish National Care Program for Breast Cancer, preoperative treatment is recommended for locally advanced or primarily inoperable breast cancer, including most T3/T4 tumors or those with fixed nodal or parasternal spread [13]. Moreover, NACT is standard for TNBC patients with tumors > 20 mm and/or nodal involvement and who are otherwise considered fit (taking comorbidities into account), consistent with the 2023 St. Gallen consensus [14]. Typical NACT regimens include at least 6 cycles of primarily anthracyclines followed by taxanes (e.g., paclitaxel or docetaxel), often combined with carboplatin and, in some cases, pembrolizumab (immunotherapy; PD-1 inhibitor) [15]. TNBC patients not achieving pCR after NACT commonly receive adjuvant capecitabine, supported by the CREATE-X trial, and, in those with germline BRCA1/2 pathogenic variants, adjuvant olaparib based on the OlympiA study [16, 17]. Retrospective studies comparing NACT and ACT in TNBC have yielded mixed results, with some studies reporting comparable survival [18–21], whereas others suggest a clinical benefit for one approach over the other [22–26]. Therefore, the benefit of postoperative therapy following NACT remains uncertain.

Given the rising use of NACT and subsequent decline in adjuvant therapy (AT; defined here as adjuvant chemotherapy [ACT] with or without adjuvant radiotherapy [ART]) alone, this study aimed to compare survival outcomes between cT1-2N0M0 TNBC patients receiving NACT ± AT or AT alone using a nationwide, population-based Swedish cohort and propensity score matching (PSM) for comparative analysis. The analysis was restricted to cT1–2N0M0 to minimize confounding by indication and compare NACT vs. ACT in a group where downstaging benefits are less decisive and both strategies are used in practice (especially around the T2 threshold). This “decision-borderline” cohort enables a fairer assessment of survival effects independent of bulky or node-positive disease.

## Methods

### Study population and data

In this population-based registry study, retrospective data were collected for 9371 samples from 9262 women diagnosed with primary, invasive TNBC either at baseline or postoperatively in Sweden between 2007 and 2021. Data on the primary tumor, including tumor stage and axillary lymph node status, as well as patient characteristics such as age and menopausal status, were provided by the Swedish National Quality Register for Breast Cancer (NBCR). Established in 2008, NBCR is a compulsory and validated Swedish quality registry that includes data on every individual diagnosed with breast cancer in Sweden [27].

Comorbidity data were sourced from the Swedish Patient Register (SPR), while information on cause of death was retrieved from the Swedish Cause of Death Register (SCDR), a mandatory national Swedish register containing information about cause of death. SCDR has been validated with a 100% coverage [28]. SPR is a mandatory registry that tracks all in- and outpatient visits within the Swedish healthcare system, recording information about diagnoses and the timing of these visits. Details about the completeness and quality of the SPR are available in Swedish [29]. Detailed definitions of tumor characteristics, treatments, comorbidities, and outcome measures are provided in the Supplementary Information.

**Fig. 1:**
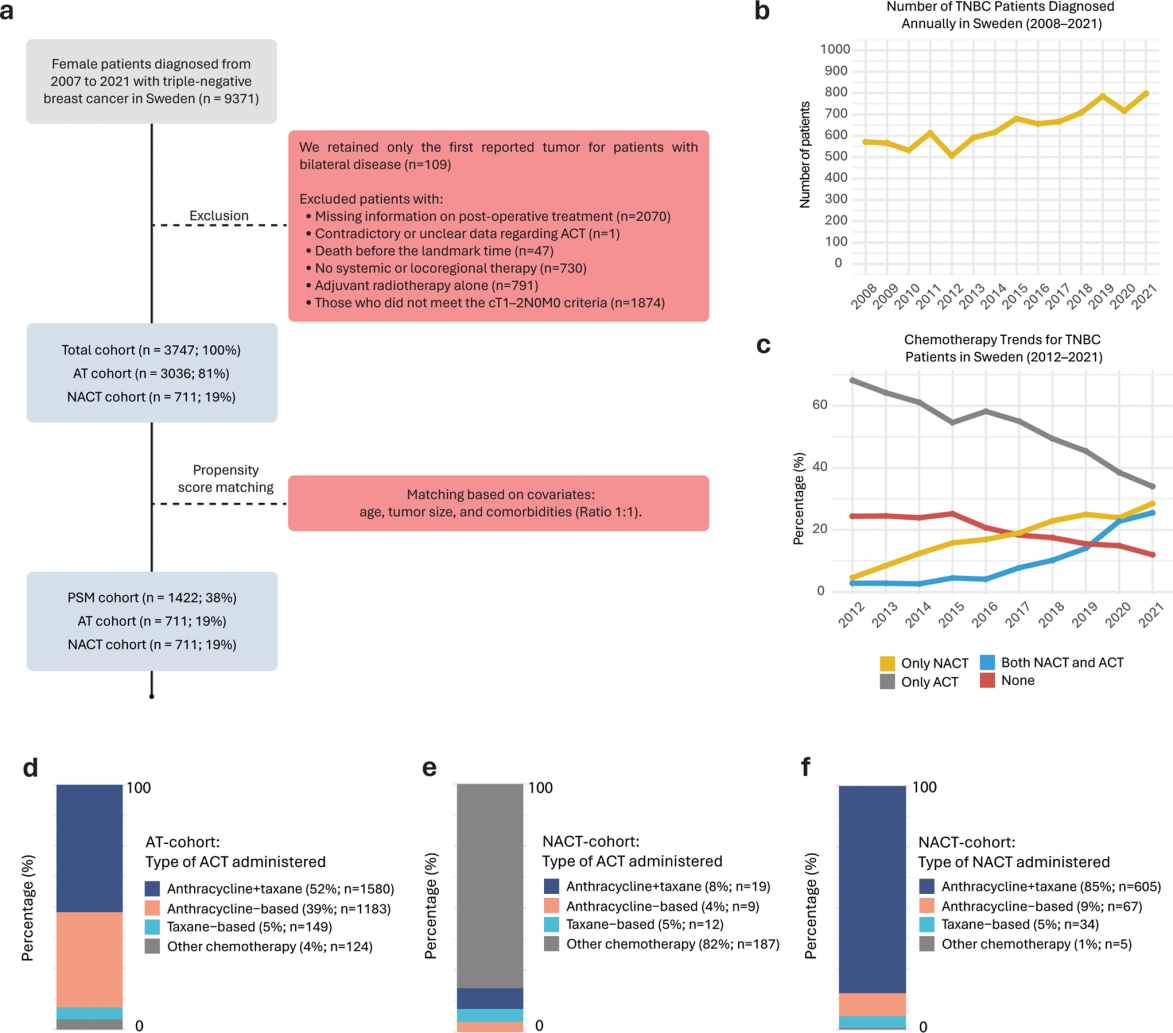
Study overview and administration of chemotherapy as neoadjuvant chemotherapy (NACT) and adjuvant chemotherapy (ACT) nationwide in Sweden. (**a**) Female patients diagnosed with triple-negative breast cancer (TNBC) in Sweden between 2007 and 2021 (*n* = 9371) were assessed for eligibility. Exclusions were made for the second tumor in bilateral cases (*n* = 109), missing information on post-operative treatment (*n* = 2070), contradictory or unclear data regarding ACT (*n* = 1), death before the landmark time (*n* = 47), no systemic or locoregional therapy (*n* = 730), adjuvant radiotherapy alone (*n* = 791), missing data or tumors staged as T0/T3/T4 (*n* = 753), nodal stage NX/N1/N2/N3 (*n* = 1011), metastatic disease (M1, *n* = 110), tumors not classified as TNBC at baseline (*n* = 2). The final cohort comprised 3747 patients, divided into two groups based on treatment: the AT cohort (*n* = 3036) and the NACT cohort (*n* = 711). Propensity score matching (PSM) was performed after excluding patients with missing data on the covariates used for matching (i.e., age, T-stage, and comorbidities). PSM was conducted using a 1:1 ratio and a caliper width of 0.2, resulting in 711 patients in each treatment group. Publicly available data from the Swedish National Quality Register for Breast Cancer for breast cancer related statistics in Sweden: (**b**) annual number of TNBC cases diagnosed in Sweden from 2008 to 2021 (mean = 650, SD = 87); (**c**) trends for chemotherapy treatment (i.e., NACT, ACT, both, or no treatment) between 2012 and 2021 for TNBC patients in Sweden. Chemotherapy administration by group: (**d**) ACT in the AT cohort; (**e**) ACT in combination with NACT in the NACT cohort; (**f**) NACT in the NACT cohort

The inclusion and exclusion criteria for the registry data are illustrated in Fig. 1A. We retained only the first reported tumor for patients with bilateral disease (*n* = 109), and excluded those with missing information on post-operative treatment (*n* = 2070), contradictory or unclear data regarding ACT (*n* = 1), death before the landmark time (*n* = 47), no systemic or locoregional therapy (*n* = 730), adjuvant radiotherapy (ART) alone (*n* = 791), and all patients who did not meet the cT1–2N0M0 criteria (*n* = 1874). The final study cohort was comprised of patients with cT1–2N0M0 TNBC at baseline, totaling 3747 individuals, stratified into the NACT cohort (*n* = 711) and the AT cohort (*n* = 3036). Data collection and handling adhered to the principles outlined in the Declaration of Helsinki and its subsequent revisions, as well as the guidelines of Good Clinical Practice. Approval for the study was obtained from the Swedish Ethical Review Authority (reference numbers 2019–05676; 2021–04421; 2022-02946-02; 2023-07905-02). Definitions of treatment cohorts, clinicopathological characteristics, staging variables, and study outcomes are described in the Supplementary Information (“Outcomes and definitions”).

### Statistical analysis

Statistical analyses were performed using R/Bioconductor (version 4.3.2) with a significance threshold set at 0.05 and two-sided p-values. Descriptive statistics were calculated utilizing the tableone R package (version 0.13.2) [30]. P-values for categorical variables (with continuity correction) and continuous variables were calculated using the Chi-square test and ANOVA, respectively. A propensity score–matched (PSM) model was constructed using nearest neighbor matching with a 1:1 ratio (one NACT-treated patient to one AT-treated patient), adjusting for age, clinical T-stage, and CCIw, carried out using the MatchIt R package (version 4.7.2) [31]. Because the study cohort was restricted to cT1–2N0M0 TNBC, confounding from nodal status, metastatic disease, and higher tumor stages was eliminated by design. Thus, age, clinical T-stage, and comorbidity were selected as the key baseline factors most likely to influence treatment allocation and survival outcomes. A caliper width of 0.2 standard deviations of the logit of the propensity score was applied to ensure comparability between matched pairs. PSM was performed using a 1:1 ratio, chosen after taking into account covariate balance, sample size, number of events, and statistical power (Supplementary Fig. 1). To account for the non-independence of observations introduced by the 1:1 PSM, Cox regression models for the PSM data were fitted with robust (sandwich) variance estimators to obtain valid standard errors and confidence intervals under the matched design.

Kaplan-Meier survival curves were generated using the survminer R package (version 0.5.0), and survival differences between groups were assessed using the log-rank test [32]. Pairwise log-rank tests were used to compare survival between groups stratified by post-treatment characteristics. To account for multiple comparisons, p-values were adjusted using the Holm method, which controls the family-wise error rate while retaining greater statistical power than the Bonferroni correction.

Univariable Cox proportional hazards models were performed to compare survival outcomes between the AT and NACT cohorts, and within each cohort when stratified by adjuvant treatment, utilizing the survival R package (version 3.7.0) [33]. Multivariable Cox regression models were adjusted for adjuvant treatment, patient age, T-stage, and comorbidities. To reduce the potential for immortal time bias associated with defining survival from the date of diagnosis rather than treatment initiation, a landmark analysis was performed using a fixed time point set at six months after diagnosis. This time point was chosen to approximate the expected window for initiating treatment, and patients who died prior to this were excluded from the study (Fig. 1A). Overall survival (OS) was defined as the time from the landmark to death from any cause, while breast cancer-specific survival (BCSS) was defined as the time from the landmark to death attributed to breast cancer. Follow-up ended at the earliest of either the date of death or the administrative cutoff (January 1, 2023). Patients who were alive or lost to follow-up at the end of the study period were censored at their last known date of survival. The analysis assumes non-informative censoring. Additional details regarding R packages are available in the Supplementary Information. Additional details on the statistical analyses and data visualizations, including the R packages used, are provided in the Supplementary Materials.

## Results

### Clinicopathological characteristics

Of the 9371 cases diagnosed with TNBC between 2007 and 2021 across the six Swedish healthcare regions, 5624 were excluded (Fig. 1A). The remaining 3747 patients were stratified into two cohorts: the NACT cohort (*n* = 711; 19%) and the AT cohort (*n* = 3036; 81%); after propensity score matching (PSM): NACT cohort (*n* = 711) and AT cohort (*n* = 711). In Sweden, the mean annual number of patients diagnosed with TNBC was 650 (SD, 87) between 2008 and 2021 (Fig. 1B).

Before PSM, patients in the AT cohort were significantly older than those in the NACT cohort (median, 59 years [IQR 48–67] vs. 53 years [IQR 43–62]; *p* < 0.001; Table 1). At baseline, 44% of patients in the NACT cohort were premenopausal, whereas 65% of patients in the AT cohort were postmenopausal (*p* < 0.001). The median follow-up time was 3.61 years (IQR 2.37–5.50) in the NACT cohort and 6.95 years (IQR 4.36–9.62) in the AT cohort. Survival status differed significantly between the AT and NACT groups both before and after PSM (both *p* < 0.001), with a higher proportion of patients alive in the NACT group (before PSM: 90.4% vs. 84.6%; after PSM: 90.4% vs. 82.4%). Because we restricted the study to cT1–2N0M0 disease, baseline stage was I–II in both cohorts; in the AT cohort, 64% of patients had stage IA and 36% stage IIA, whereas in the NACT cohort 18% had stage IA and 82% stage IIA. Tumors were generally larger in the NACT cohort, with a higher proportion of T2 tumors (82%) compared to the AT cohort (36%; *p* < 0.001). Most patients had no comorbidities (CCIw = 0), with a higher proportion in the NACT cohort compared to the AT cohort (82% vs. 75%; *p* < 0.001).

### Treatment characteristics

The proportion of patients receiving NACT increased over time, with the majority diagnosed from 2015 onward, whereas patients in the AT cohort were more evenly distributed across the study period (*p* < 0.001; Fig. 1C; Table 1). In the AT cohort, 74% of patients received both ACT and ART, and 26% received ACT alone (Supplementary Table 1). Most patients in the NACT cohort also received adjuvant treatment, with 60% receiving ART, 25% receiving both ACT + ART, and 7% ACT alone (Supplementary Table 2). In the AT cohort, the most commonly administered form of ACT was a combination of anthracycline and taxane (52%), followed by anthracycline-based monotherapy (39%), with 77% of patients completing ACT according to plan (Table 1; Fig. 1D). Side effects were the most commonly reported reason for treatment discontinuation (19%), with reasons unknown in 78% of cases due to missing data (Table 1).

In the NACT cohort, only a minority received ACT (32%), most often recorded as an unknown regimen (26%), while anthracycline-based (1.3%), combination anthracycline–taxane (2.7%), and taxane-based monotherapy (1.7%) were rare. The majority (68%) received no additional chemotherapy postoperatively (Table 1; Fig. 1E). In this cohort, the most commonly administered neoadjuvant regimen was a combination of anthracycline and taxane (85%), followed by anthracycline-based (9.4%) and taxane-based (4.8%) monotherapy, while the regimen was unknown in 0.7% of patients (Table 1; Fig. 1F). In total, 67% completed treatment according to plan, while side effects were the most common reason for discontinuation (25%); however, data on reasons for discontinuation were missing for 68% of patients (Table 1). Breast-conserving surgery was common in both the AT (69%) and NACT (65%) cohorts, as well as mastectomy (31% vs. 33%; Table 1). Sentinel node biopsy alone was more frequently performed in the AT cohort (82% vs. 70%), while axillary lymph node dissection alone was more common in the NACT cohort (13% vs. 3.8%; Table 1). ART was more often administered in the NACT cohort (82%) than in the AT cohort (74%). Regarding locoregional treatment, BCS with ART was the most common approach in both cohorts (65% AT vs. 63% NACT), followed by mastectomy alone (21% AT vs. 15% NACT) and mastectomy in combination with radiotherapy (9.5% AT vs. 18% NACT; Table 1).

### Pathological complete response to neoadjuvant chemotherapy

**Fig. 2:**
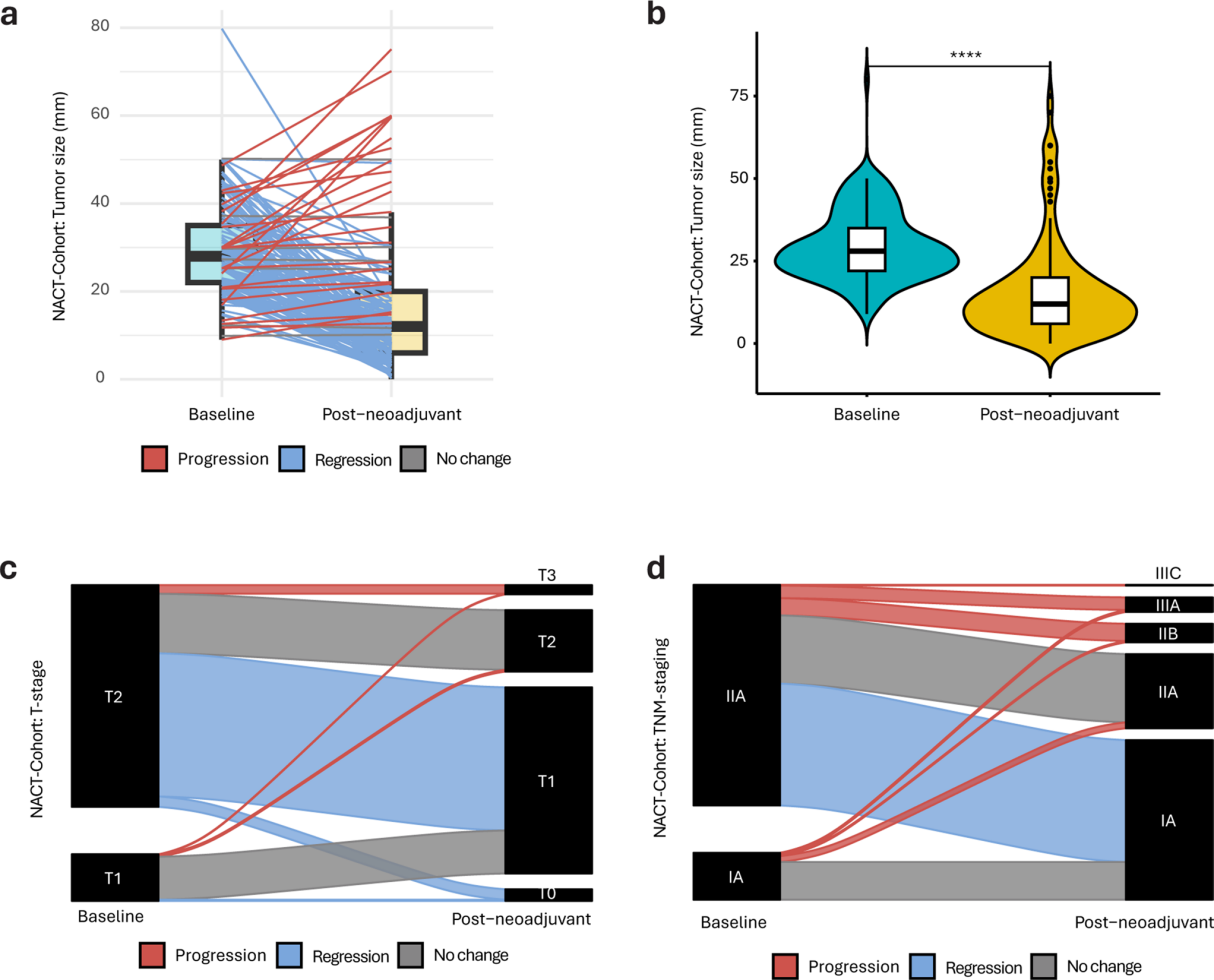
Neoadjvuant chemotherapy (NACT) leads to tumor regression. (**a**) Box plot in combination with a line plot, before-after for tumor size (mm), depicting differences from baseline (left) to post-surgery (right) for patients who received NACT. Only patients who had complete measurements at baseline and post-surgery were included. Blue indicates tumor regression, red for progression, and gray depicts no change in tumor size. (**b**) Violin plot for the same tumor size data. Turquoise indicates baseline (left) and yellow depicts post-surgery (right). T-test was used to calculate statistically significant differences in tumor size between baseline and post-surgery. *p < 0.05; **p ≤ 0.01; ***p ≤ 0.001; ****p ≤ 0.0001. Sankey plot for (**c**) tumor staging (T1-2) and (**d**) TNM staging at baseline (left) and post-surgery (right) for patients receiving NACT. Red indicates tumor progression, blue indicates regression, and gray depicts no change

Tumor size was significantly reduced following NACT (*p* < 0.0001; Fig. 2A–B), with a reduction in median tumor diameter from 27 mm (IQR, 22–35) at baseline to 13 mm (IQR, 6–21) post-surgery (Table 1). Following NACT, 58% of patients experienced downstaging in T-stage, with 38% having no change and 4.1% showing pathological progression (Fig. 2C). Following NACT, 46% of patients experienced downstaging in TNM stage, 40% had no change, and 15% showed pathological progression (Fig. 2D). Following NACT and surgery, 684 samples (96%) were still TNBC. The remaining 27 tumors were reassigned to luminal A (*n* = 15; 2.1%), luminal B/HER2− (*n* = 6; 0.8%), non-luminal HER2+ (*n* = 5; 0.7%), or ER−/PR+/HER2− (*n* = 1; 0.1%; Table 1).

### Survival analysis before and after PSM between the AT and NACT cohorts

**Fig. 3:**
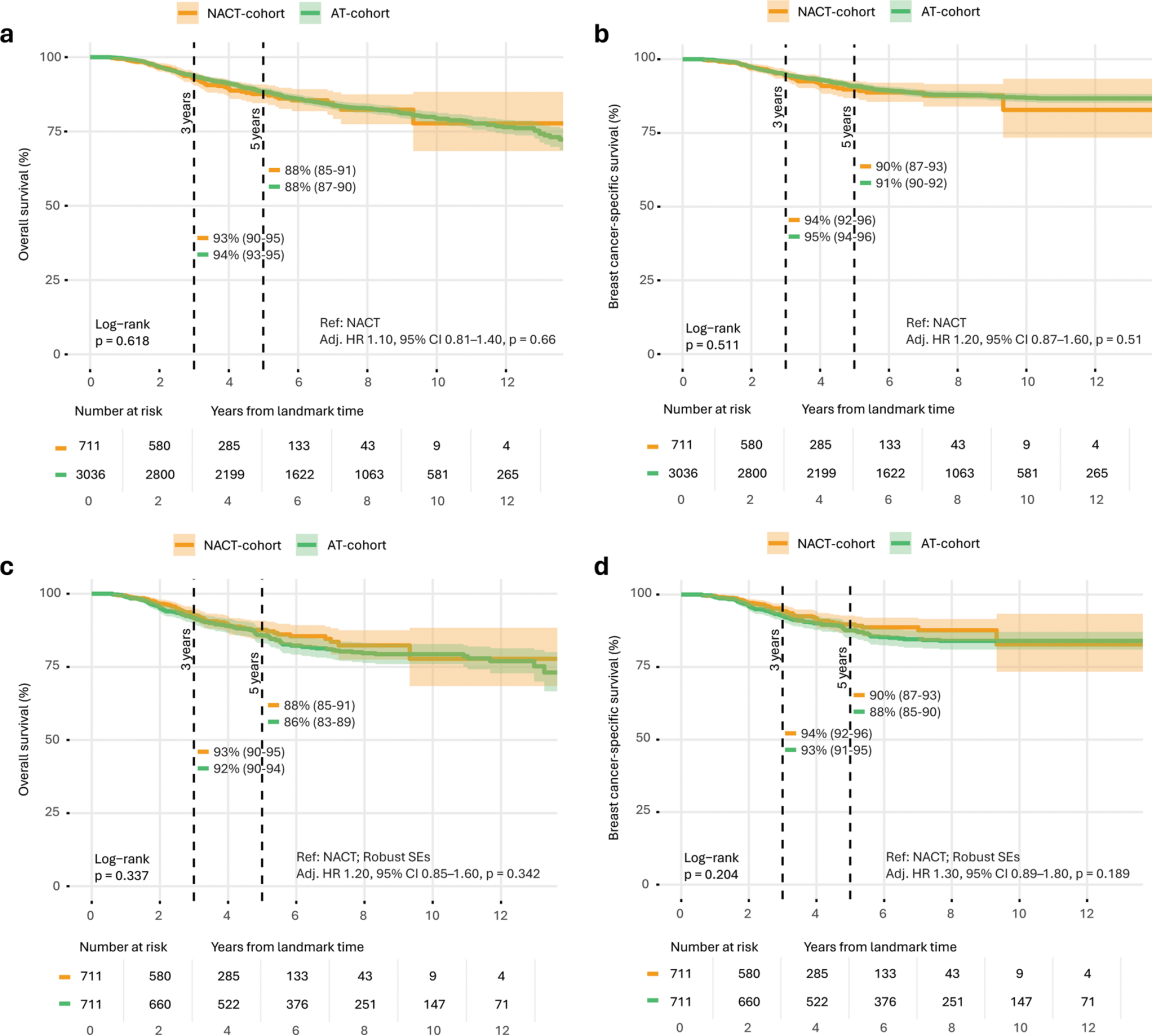
Kaplan–Meier plots comparing the NACT and AT cohorts before and after PSM. Dotted vertical black lines indicate median 3- (left) and 5-year survival (right). Shaded areas represent 95% confidence intervals. Each interval on the x-axis represents 2 years, with a total visualized follow-up time of 14 years. Adjusted hazard ratio (HR) estimates from multivariable Cox regression analysis, adjusted for clinical T-stage, age, and comorbidities, are shown with NACT as the reference group. (**a**) Overall survival (OS) and (**b**) breast cancer-specific survival (BCSS) for the NACT cohort (orange) versus the AT cohort (green) before propensity score matching (PSM). (**c**) OS and (**d**) BCSS for the NACT cohort versus the AT cohort after PSM. *p < 0.05; **p ≤ 0.01; ***p ≤ 0.001; ****p ≤ 0.0001

Kaplan–Meier analysis showed comparable OS and BCSS between the AT and NACT cohorts prior to PSM, with similar 5-year OS (88%) and BCSS (90–91%) probabilities in both cohorts (Fig. 3A–B). Log-rank tests confirmed these similarities (OS: *p* = 0.62; BCSS: *p* = 0.51), and both uni- and multivariable Cox regression analyses indicated no significant differences in risk of death between the groups. However, these unadjusted comparisons could potentially reflect baseline imbalances between groups. After 1:1 PSM, 711 patients remained in each treatment group (Supplementary Fig. 1). Median BCSS follow-up in the matched cohort was 3.61 years (IQR 2.37–5.50) for the NACT cohort and 7.25 years (IQR 4.73–9.94) for the AT cohort. Although patient and tumor characteristics were well-balanced between the groups for the covariates selected in the PSM, differences remained in surgical procedures, adjuvant treatment strategies, and other factors (Table 1). Following PSM, survival estimates remained comparable (Fig. 3C–D), with similar 5-year OS (NACT: 88%; AT: 86%; log-rank *p* = 0.34) and BCSS (NACT: 90%; AT: 86%; log-rank *p* = 0.20). These findings indicate that after matching, survival outcomes remained comparable between the two cohorts. In the NACT cohort, the most common cause of non–breast cancer-related death was not reported (56%), followed by neoplasms (11%) and abnormal clinical findings (11%), while in the AT cohort, not reported was also most common (32%), followed by neoplasms (30%; Supplementary Fig. 2A–B).

### Survival analysis stratified by adjuvant treatment

**Fig. 4:**
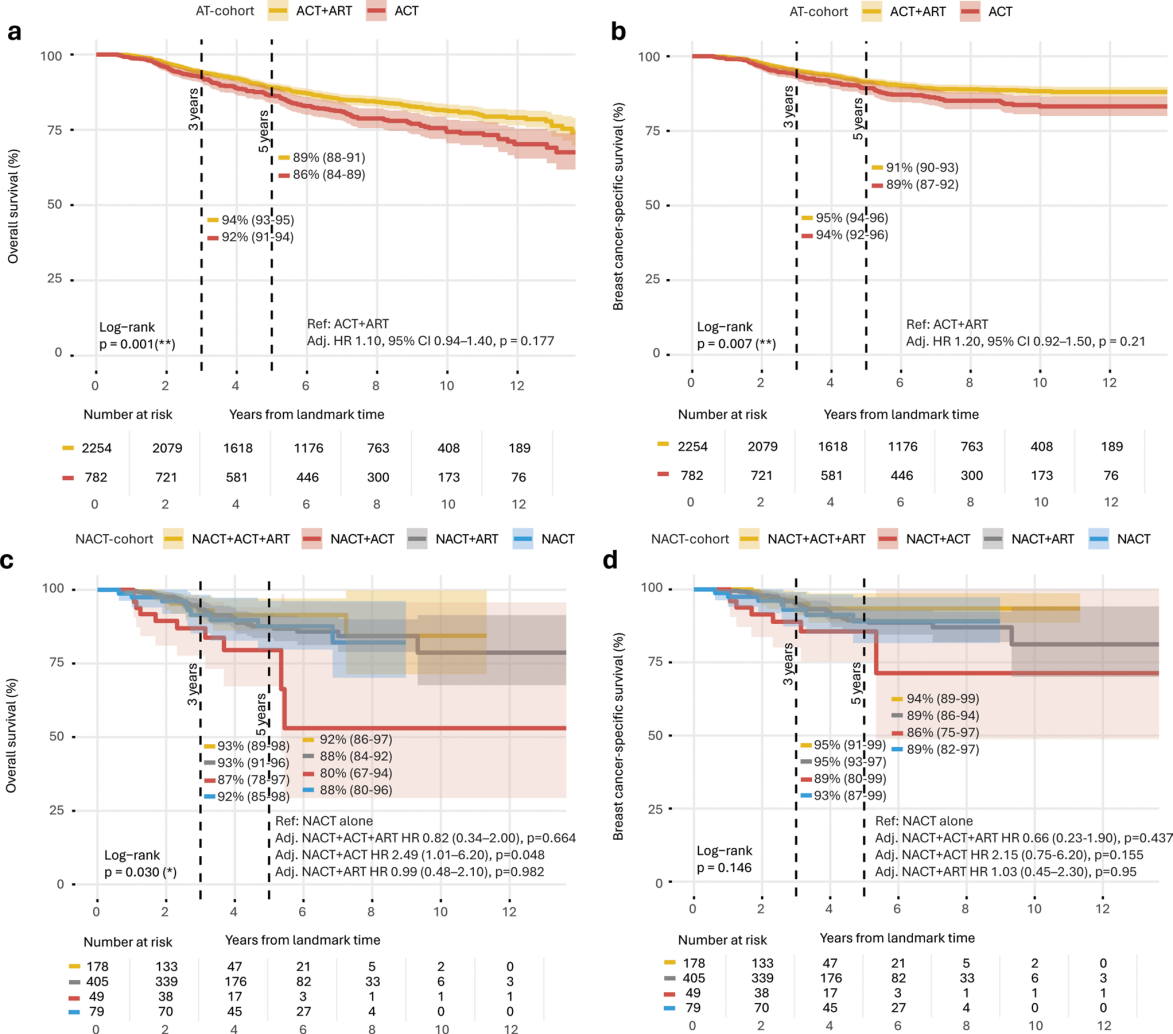
Kaplan–Meier plots on the NACT and AT cohorts stratified by adjuvant treatment. Dotted vertical black lines indicate median 3- (left) and 5-year survival (right). Shaded areas represent 95% confidence intervals (95% CI). Each interval on the x-axis represents 2 years, with a total visualized follow-up time of 14 years. Adjusted hazard ratio (HR) estimates from multivariable Cox regression analysis, adjusted for clinical T-stage, age, and comorbidities, are shown with ACT + ART combined as the reference group for the AT cohort, and no adjuvant treatment (i.e., NACT alone) as the reference group for the NACT cohort. (**a**) Overall survival (OS) and (**b**) breast cancer-specific survival (BCSS) for the AT cohort stratified by adjuvant treatment (i.e., ACT + ART, ART, or ACT). (**c**) OS and (**d**) BCSS for the NACT cohort stratified by AT (i.e., NACT + ART+ACT, NACT + ART, NACT + ACT, or only NACT). *p < 0.05; **p ≤ 0.01; ***p ≤ 0.001; **p ≤ 0.0001

In the AT cohort, patients receiving ACT alone had more unfavorable OS and BCSS than those given ACT + ART (Fig. 4A–B and Supplementary Table 1). For OS, the 5-year survival rate was 89% (95% CI, 88–91) in the ACT + ART group compared to 86% (95% CI, 84–89) in the ACT group (log-rank *p* = 0.00114). Using ACT + ART as the reference, univariable Cox regression showed a higher risk of death in the ACT group (*p* = 0.001). However, this association was not statistically significant in the multivariable analysis (*p* = 0.177). For BCSS, the corresponding 5-year survival rates were 91% (95% CI, 90–93) for ACT + ART and 89% (95% CI, 87–92) for ACT (log-rank *p* = 0.00723). Again, ACT was associated with a higher risk of breast cancer death compared to ACT + ART (*p* = 0.007). However, this association was also not statistically significant in the multivariable analysis (*p* = 0.21; Fig. 4A–B).

In the NACT cohort, survival outcomes varied according to additional adjuvant treatment (Fig. 4C–D and Supplementary Table 2). The best 5-year OS was seen with NACT + ACT+ART (92%), and the lowest with NACT + ACT (80%); the remaining groups were intermediate at 88% (global log-rank *p* = 0.030). Compared to NACT alone, multivariable Cox regression showed no significant differences for NACT + ACT+ART (*p* = 0.66) or NACT + ART (*p* = 0.98), whereas NACT + ACT was associated with a higher risk of death (HR 2.49; 95% CI: 1.01–6.2; *p* = 0.048). For BCSS, the 5-year survival rates were 94% (95% CI: 89–99) for NACT + ACT+ART, 89% (86–94) for NACT + ART, 86% (75–97) for NACT + ACT, and 89% (82–97) for NACT alone (log-rank *p* = 0.146). In the multivariable Cox regression analysis, none of the comparisons with NACT alone reached statistical significance (Fig. 4C–D).

### Survival analysis stratified by multimodality treatment

When stratified by multimodality treatment in the AT cohort, significant differences in OS and BCSS were observed across groups (log-rank *p* < 0.0001 for both; Supplementary Fig. 3). The highest 5-year OS was seen in the ACT + BCS+ART group (91%; 95% CI, 90–93), while the lowest was in the ACT+Mastectomy + ART group (76%; 95% CI, 71–81). In multivariable analysis using ACT+Mastectomy + ART as the reference, both ACT+Mastectomy alone (HR 0.57; 95% CI: 0.44–0.76; *p* < 0.001) and ACT + BCS+ART (HR 0.48; 95% CI: 0.37–0.62; *p* < 0.001) were independently associated with lower mortality. Similar patterns were observed for BCSS (Supplementary Fig. 4).

In the NACT cohort, 5-year OS ranged from 73% (Mastectomy + ART alone) to 92% (ACT + BCS+ART and BCS + ART alone; log-rank *p* < 0.0001; Supplementary Fig. 5). In multivariable analysis, no multimodality treatment group differed significantly from the ACT+Mastectomy + ART reference for OS. For BCSS, ACT + BCS alone was associated with a higher risk (HR 7.7; 95% CI: 1.24–48.2; *p* = 0.028), though this group was very small (*n* = 6) and should be interpreted with caution (Supplementary Fig. 6).

## Discussion

This Swedish nationwide registry-based study retrospectively examined the survival outcomes of female patients diagnosed with cT1–2N0M0 TNBC who underwent NACT ± AT compared with AT alone. Before propensity score matching, both OS and BCSS showed comparable results. However, substantial differences in patient- and tumor-related characteristics were observed between the cohorts. After matching, survival outcomes remained similar between the groups, suggesting that the observed comparability was not explained by baseline imbalances, but rather reflected a true lack of difference in survival once confounding by indication had been minimized.

An individual patient-level meta-analysis of randomized evidence from the Early Breast Cancer Trialists’ Collaborative Group (EBCTCG) reported comparable distant recurrence, breast cancer mortality, and overall mortality between NACT and ACT, but a higher risk of local recurrence following NACT [12]. However, these findings were largely derived from older randomized trials with variable treatment protocols, making it difficult to draw firm conclusions for patients with triple-negative breast cancer in current clinical practice. This underscores the value of modern population-based studies in assessing how treatment sequencing influences survival in this subgroup.

These findings are in line with earlier reports, notably the European Organization for Research and Treatment of Cancer (EORTC) trial 10,902, which observed similar survival outcomes in breast cancer patients treated with NACT and AT [34]. However, in landmark randomized trials such as NSABP B-18 and EORTC 10,902, systemic chemotherapy was delivered entirely preoperatively in the NACT arm and entirely postoperatively in the adjuvant arm. In contrast, in our real-world cohort from 2007 to 2021, most patients in the NACT group also received additional ACT and/or ART, reflecting treatment practice during that period and potentially influencing survival outcomes.

An interesting finding from our study is the influence of NACT on surgical management, which shows some potential for de-escalation but appears inconsistently applied. Before PSM, mastectomy was equally common between the cohorts, and ALND was more common among patients receiving NACT, reflecting the selection of individuals with more advanced disease for this treatment approach. After PSM, mastectomy rates were significantly higher in the AT group, whereas breast-conserving surgery was more common in the NACT cohort, suggesting that NACT may have facilitated less extensive breast surgery. In contrast, ALND continued to be performed more frequently in the NACT group, both before and after PSM. In line with the observations by Tinterri et al. [35], these findings suggest that while NACT may support breast-conserving surgery, its potential to achieve consistent surgical de-escalation, particularly at the axillary level, has not been fully realized in clinical practice for this patient cohort.

Consistent with this, survival outcomes in both cohorts varied markedly by multimodality treatment group. In the AT cohort, patients treated with ACT + BCS+ART had the most favorable OS and BCSS, while those receiving ACT+Mastectomy + ART, the reference group, had the worst outcomes, likely reflecting that mastectomy was preferentially performed in patients with larger or more centrally located tumors. After multivariable adjustment, both ACT+Mastectomy alone and ACT + BCS+ART were independently associated with lower mortality compared to ACT+Mastectomy + ART, suggesting that the apparent survival differences are at least partly attributable to patient selection rather than a direct effect of surgical approach or radiotherapy. In the NACT cohort, where group sizes were considerably smaller, no multimodality treatment group differed significantly from the reference in multivariable analysis, with the exception of ACT + BCS alone for BCSS, a finding that should be interpreted cautiously given the very small number of patients in that group (*n* = 6).

Patients who received a combination of NACT and ACT ± ART tended to present with larger tumors at baseline compared with the other treatment groups. In many cases, nodal disease to ypN1 after NACT, a finding consistent with previous studies showing that postoperative systemic therapy and radiotherapy following NACT is most often given to patients with residual nodal disease or other high-risk pathological features [16, 36]. These factors likely explain the worse survival in the NACT + ACT group, rather than suggesting a detrimental effect of additional postoperative therapy.

The higher mastectomy rate observed in the NACT + ACT group may, in part, reflect differences in baseline tumor-to-breast volume ratio, which is a key determinant of surgical approach, as well as temporal changes in surgical practice during the study period. In the earlier years after the introduction of NACT, surgeons were generally more cautious in downscaling surgery according to treatment response, and mastectomy was therefore chosen more frequently than in later years, where breast-conserving surgery was increasingly preferred whenever feasible. These patterns are in line with prior observations that surgical decision-making after NACT is influenced both by tumor response in the breast and by the extent of residual axillary disease, the latter being a key determinant of radiotherapy and ALND indications [37].

Our findings should be interpreted in the context of both the strengths and limitations of the study design and data sources. When treatment strategies are compared in real-world settings without random allocation, as in this study where assignment to NACT or AT was based on clinical judgment and evaluated retrospectively, the inherent risk of indication bias can substantially complicate the interpretation of the findings [38]. Although we applied propensity score matching to reduce this risk and strengthen the validity of our results, residual confounding is still likely. This persistent difference after matching may indicate residual prognostic imbalance between the groups, and because survival status at last follow-up is a crude descriptive measure, it should not be interpreted as a causal survival advantage of NACT. Furthermore, existing evidence suggests that survival outcomes are often similar with neoadjuvant versus adjuvant sequencing in early-stage TNBC, such that sequencing decisions may be driven primarily by goals of downstaging and response assessment rather than expectations of improved long-term survival. Importantly, NACT is associated with practical risks, including treatment-related toxicity or delays to definitive surgery, which may be particularly impactful for patients of advanced age or with substantial comorbidity. Moreover, the NACT cohort was not exclusively treated with NACT, as many also received postoperative therapy, which should be considered to avoid interpreting this study as a direct comparison of NACT versus AT alone.

This study is limited by the fact that systemic therapy regimens reflect the 2007–2021 treatment era and are not fully representative of contemporary TNBC standards. Because our cohort was treated between 2007 and 2021, it does not capture the subsequent adoption of pembrolizumab-based therapy for high-risk early-stage TNBC in Sweden. In light of the improved event-free and overall survival reported in KEYNOTE-522, survival outcomes in a present-day TNBC cohort could therefore differ from those reported in this study [39]. In addition, postoperative chemotherapy after NACT could not be characterized in detail because the registry captures postoperative regimens using broad categories, with a substantial proportion coded as ‘other,’ limiting interpretation of response-guided adjuvant treatment. Similarly, full pathologic complete response after NACT could not be evaluated because the registry did not include a validated pCR variable, and derivation from incomplete ypT and ypN data may have introduced misclassification. Moreover, contemporary TNBC management increasingly uses strategies after neoadjuvant therapy in which residual disease can lead to escalation or modification of postoperative systemic treatment, but because these approaches were not uniformly implemented during much of our study period and could not be evaluated in detail in our registry data, our results mainly reflect an earlier treatment era and may not fully generalize to current practice where postoperative therapy is tailored based on residual disease. Some variables had missing values, which were generally infrequent and evenly distributed between the treatment groups. Missing data were handled by complete-case analysis, and no imputation was performed. Nevertheless, the presence of missing information may have introduced bias and should be considered when interpreting the findings.

To conclude, our Swedish population registry study shows similar long term survival between neoadjuvant and adjuvant chemotherapy in an era largely preceding widespread adoption of contemporary regimens and response guided postoperative escalation. In this cohort, chemotherapy sequencing was not associated with a detectable survival advantage.

**Table 1:** Clinicopathological characteristics and treatment details of 3747 TNBC patients, stratified by the AT cohort, before and after PSM

Characteristics	Data before PSM			Data after PSM		
	AT (*n* = 3036)	NACT (*n* = 711)	*p*-value	AT (*n* = 711)	NACT (*n* = 711)	*p*-value
**Patient age at baseline**,** years (median [IQR])**	**59.00 [48.00**,**67.00]**	**53.00 [43.00**,**62.00]**	**< 0.001**	**53.00 [43.00**,**62.00]**	**53.00 [43.00**,**62.00]**	**0.783**
Tumor size (mm) at baseline (median [IQR])	16.00 [12.00,31.00]	27.00 [22.00,35.00]	0.052	31.00 [26.50,34.50]	27.00 [22.00,35.00]	0.633
Tumor size (mm) post-surgery (median [IQR])	19.00 [13.50,25.00]	13.00 [6.00,21.00]	< 0.001	25.00 [19.00,31.00]	13.00 [6.00,21.00]	< 0.001
Ki67% at baseline (median [IQR])	40.00 [30.00,75.00]	70.00 [49.00,83.00]	0.086	40.00 [22.00,40.00]	70.00 [49.00,83.00]	0.019
Ki67% post-surgery (median [IQR])	68.00 [42.00,82.00]	47.00 [12.00,80.00]	< 0.001	70.00 [46.00,85.00]	47.00 [12.00,80.00]	< 0.001
Swedish healthcare region, n (%)						
Mid Sweden	597 (19.7)	63 (8.9)	< 0.001	137 (19.3)	63 (8.9)	< 0.001
North	272 (9.0)	26 (3.7)		42 (5.9)	26 (3.7)	
South	512 (16.9)	193 (27.1)		142 (20.0)	193 (27.1)	
Southeast	336 (11.1)	80 (11.3)		110 (15.5)	80 (11.3)	
Stockholm/Gotland	551 (18.1)	255 (35.9)		154 (21.7)	255 (35.9)	
West	768 (25.3)	94 (13.2)		126 (17.7)	94 (13.2)	
Age range at diagnosis, years, n (%)						
< 40	307 (10.1)	124 (17.4)	< 0.001	126 (17.7)	124 (17.4)	0.99
40–49	533 (17.6)	178 (25.0)		171 (24.1)	178 (25.0)	
50–64	1178 (38.8)	266 (37.4)		271 (38.1)	266 (37.4)	
65–74	826 (27.2)	115 (16.2)		113 (15.9)	115 (16.2)	
>=75	192 (6.3)	28 (3.9)		30 (4.2)	28 (3.9)	
Year of diagnosis (%)						
2007–2008	56 (1.8)	3 (0.4)	< 0.001	13 (1.8)	3 (0.4)	< 0.001
2009–2010	295 (9.7)	5 (0.7)		76 (10.7)	5 (0.7)	
2011–2012	411 (13.5)	8 (1.1)		106 (14.9)	8 (1.1)	
2013–2014	565 (18.6)	41 (5.8)		142 (20.0)	41 (5.8)	
2015–2016	576 (19.0)	105 (14.8)		131 (18.4)	105 (14.8)	
2017–2018	512 (16.9)	162 (22.8)		125 (17.6)	162 (22.8)	
2019–2020	478 (15.7)	276 (38.8)		100 (14.1)	276 (38.8)	
2021	143 (4.7)	111 (15.6)		18 (2.5)	111 (15.6)	
Menopausal status at baseline (%)						
Premenopausal	836 (27.5)	313 (44.0)	< 0.001	285 (40.1)	313 (44.0)	0.134
Postmenopausal	1955 (64.4)	355 (49.9)		367 (51.6)	355 (49.9)	
Missing data	245 (8.1)	43 (6.0)		59 (8.3)	43 (6.0)	
**Clinical T stage (cT; %)**						
T1	1946 (64.1)	127 (17.9)	< 0.001	126 (17.7)	127 (17.9)	1
T2	1090 (35.9)	584 (82.1)		585 (82.3)	584 (82.1)	
Clinical stage (cTNM)(%)						
IA	1946 (64.1)	127 (17.9)	< 0.001	126 (17.7)	127 (17.9)	1
IIA	1090 (35.9)	584 (82.1)		585 (82.3)	584 (82.1)	
Pathological subtype (%)						
TNBC	3036 (100)	684 (96.2)	< 0.001	711 (100.0)	684 (96.2)	< 0.001
Luminal A	0 (0.0)	15 (2.1)		0 (0.0)	15 (2.1)	
Luminal B/HER2-	0 (0.0)	6 (0.8)		0 (0.0)	6 (0.8)	
Non-luminal HER2+	0 (0.0)	5 (0.7)		0 (0.0)	5 (0.7)	
Unspecified (ER-,PR+,HER2-)	0 (0.0)	1 (0.1)		0 (0.0)	1 (0.1)	
Pathological T stage (ypT; %)						
T0	1 (0.0)	19 (2.7)	< 0.001	0 (0.0)	19 (2.7)	< 0.001
T1	1796 (59.2)	303 (42.6)		237 (33.3)	303 (42.6)	
T2	1182 (38.9)	100 (14.1)		455 (64.0)	100 (14.1)	
T3	44 (1.4)	15 (2.1)		16 (2.3)	15 (2.1)	
Missing data	13 (0.4)	274 (38.5)		3 (0.4)	274 (38.5)	
Pathological N stage (ypN; %)						
N0	2391 (78.8)	557 (78.3)	< 0.001	531 (74.7)	557 (78.3)	< 0.001
N1	525 (17.3)	99 (13.9)		148 (20.8)	99 (13.9)	
N2	48 (1.6)	19 (2.7)		15 (2.1)	19 (2.7)	
N3	25 (0.8)	2 (0.3)		6 (0.8)	2 (0.3)	
NX	47 (1.5)	34 (4.8)		11 (1.5)	34 (4.8)	
Pathological stage (ypTNM)(%)						
IA	1517 (50.0)	241 (33.9)	< 0.001	192 (27.0)	241 (33.9)	< 0.001
IIA	1080 (35.6)	112 (15.8)		371 (52.2)	112 (15.8)	
IIB	290 (9.6)	28 (3.9)		106 (14.9)	28 (3.9)	
IIIA	65 (2.1)	22 (3.1)		22 (3.1)	22 (3.1)	
IIIC	25 (0.8)	1 (0.1)		6 (0.8)	1 (0.1)	
Unspecified	59 (1.9)	307 (43.2)		14 (2.0)	307 (43.2)	
NHG (%)						
Grade1	27 (0.9)	10 (1.4)	< 0.001	5 (0.7)	10 (1.4)	< 0.001
Grade2	434 (14.3)	136 (19.1)		82 (11.5)	136 (19.1)	
Grade3	2537 (83.6)	204 (28.7)		616 (86.6)	204 (28.7)	
Missing data	38 (1.3)	361 (50.8)		8 (1.1)	361 (50.8)	
Survival status (%)						
Alive	2567 (84.6)	643 (90.4)	< 0.001	586 (82.4)	643 (90.4)	< 0.001
Death by BC	286 (9.4)	50 (7.0)		92 (12.9)	50 (7.0)	
Death by other causes	183 (6.0)	18 (2.5)		33 (4.6)	18 (2.5)	
**Charlson comorbidity index**,** weighted**,** grouped (%)**						
CCIw = 0	2268 (74.7)	583 (82.0)	< 0.001	593 (83.4)	583 (82.0)	0.675
CCIw = 1–3	721 (23.7)	126 (17.7)		115 (16.2)	126 (17.7)	
CCIw = 4–10	47 (1.5)	2 (0.3)		3 (0.4)	2 (0.3)	
Surgery (%)						
Mastectomy	928 (30.6)	232 (32.6)	< 0.001	300 (42.2)	232 (32.6)	< 0.001
Breast-conserving surgery	2093 (68.9)	460 (64.7)		404 (56.8)	460 (64.7)	
Subcutaneous mastectomy	11 (0.4)	17 (2.4)		5 (0.7)	17 (2.4)	
Only axilla surgery	1 (0.0)	0 (0.0)		0 (0.0)	0 (0.0)	
Missing data	3 (0.1)	2 (0.3)		2 (0.3)	2 (0.3)	
Axillary surgery (%)						
SN	2494 (82.1)	500 (70.3)	< 0.001	536 (75.4)	500 (70.3)	< 0.001
ALND	116 (3.8)	93 (13.1)		44 (6.2)	93 (13.1)	
SN and ALND	366 (12.1)	102 (14.3)		119 (16.7)	102 (14.3)	
Sampling	45 (1.5)	0 (0.0)		9 (1.3)	0 (0.0)	
Missing data	15 (0.5)	16 (2.3)		3 (0.4)	16 (2.3)	
Neoadjuvant chemotherapy (%)						
Yes	0 (0.0)	711 (100.0)	< 0.001	0 (0.0)	711 (100.0)	< 0.001
No	3036 (100.0)	0 (0.0)		711 (100.0)	0 (0.0)	
Completed NACT according to plan (%)						
Yes	0 (0.0)	477 (67.1)	< 0.001	0 (0.0)	477 (67.1)	< 0.001
No	0 (0.0)	231 (32.5)		0 (0.0)	231 (32.5)	
Missing data	3036 (100.0)	3 (0.4)		711 (100.0)	0 (0.0)	
Reason for discontinuation of NACT (%)						
Side effects	0 (0.0)	177 (24.9)	< 0.001	0 (0.0)	177 (24.9)	< 0.001
Other	0 (0.0)	53 (7.5)		0 (0.0)	53 (7.5)	
Missing data	3036 (100.0)	481 (67.6)		711 (100.0)	481 (67.6)	
NACT administered (%)						
Anthracycline-based	0 (0.0)	67 (9.4)	< 0.001	0 (0.0)	67 (9.4)	< 0.001
Anthracycline+taxane	0 (0.0)	605 (85.1)		0 (0.0)	605 (85.1)	
Taxane-based	0 (0.0)	34 (4.8)		0 (0.0)	34 (4.8)	
Other chemotherapy	0 (0.0)	5 (0.7)		0 (0.0)	5 (0.7)	
None	3036 (100.0)	0 (0.0)		711 (0.0)	0 (0.0)	
Adjuvant chemotherapy (%)						
Yes	3036 (100.0)	227 (31.9)	< 0.001	711 (100.0)	227 (31.9)	< 0.001
No	0 (0.0)	484 (68.1)		0 (0.0)	484 (68.1)	
Completed ACT according to plan (%)						
Yes	2351 (77.4)	143 (20.1)	< 0.001	551 (77.5)	143 (20.1)	< 0.001
No	666 (21.9)	81 (11.4)		152 (21.4)	81 (11.4)	
Missing data	19 (0.7)	487 (68.5)		8 (1.1)	487 (68.5)	
Reason for discontinuation of ACT (%)						
Side effects	566 (18.6)	62 (8.7)	< 0.001	128 (18.0)	62 (8.7)	
Other	93 (3.1)	18 (2.5)		22 (3.1)	18 (2.5)	
Missing data	2377 (78.3)	631 (88.7)		561 (78.9)	631 (88.7)	
ACT administered (%)						
Anthracycline-based	1183 (39.0)	9 (1.3)	< 0.001	291 (40.9)	9 (1.3)	< 0.001
Anthracycline+taxane	1580 (52.0)	19 (2.7)		369 (51.9)	19 (2.7)	
Taxane-based	149 (4.9)	12 (1.7)		23 (3.2)	12 (1.7)	
Other chemotherapy	124 (4.1)	187 (26.3)		28 (3.9)	187 (26.3)	
None	0 (0.0)	484 (68.1)		0 (0.0)	484 (68.1)	
Adjuvant radiotherapy (%)						
Yes	2254 (74.2)	583 (82.0)	< 0.001	476 (66.9)	583 (82.0)	< 0.001
No	782 (25.8)	128 (18.0)		235 (33.1)	128 (18.0)	
Locoregional treatment (%)						
BCS + ART	1960 (64.6)	450 (63.3)	< 0.001	375 (52.7)	450 (63.3)	< 0.001
Mastectomy only	641 (21.1)	104 (14.6)		202 (28.4)	104 (14.6)	
Masectomy + ART	287 (9.5)	128 (18.0)		98 (13.8)	128 (18.0)	
Other	148 (4.9)	29 (4.1)		36 (5.1)	29 (4.1)	

P-values were calculated using Chi-square test for categorical variables (with continuity correction) and ANOVA for continuous variables. Variables in bold were included in the propensity score matching. Abbreviations: ACT = Adjuvant chemotherapy; ALND = Axillary lymph node dissection; ANOVA = Analysis of variance; ART = Adjuvant radiotherapy; AT = Adjuvant therapy; BC = Breast cancer; BCS = Breast-conserving surgery; CCIw = Charlson Comorbidity Index, weighted; ER = Estrogen receptor; HER2 = Human epidermal growth factor receptor2; IQR = Interquartile range; NACT = Neoadjuvant chemotherapy; NHG = Nottingham histologic grade; NX = Nodal status unknown; PR = Progesterone receptor; PSM = Propensity score matching; SN = Sentinel node; TNBC = Triple-negative breast cancer

## Supplementary Information

Below is the link to the electronic supplementary material.

Supplementary Material 1

Supplementary Material 2

## Data Availability

The data are unavailable for public access due to Swedish and European legal restrictions aimed at protecting patient privacy. However, researchers with ethical approvals who meet the criteria for accessing confidential information may request data through the Swedish National Quality Register for Breast Cancer.
