# Supplementary material for: Retrospective study of comparable survival after neoadjuvant versus adjuvant chemotherapy in cT1-2N0M0 triple-negative breast cancer
Source: Breast Cancer Res Treat. 2026 May 12;217(2):34. doi: 10.1007/s10549-026-07983-9 (PMC13167834; doi:10.1007/s10549-026-07983-9)
Supplement: Supplementary file 1 — Supplementary Material 1 [file 10549_2026_7983_MOESM1_ESM.docx]

**Supplementary information**

**Retrospective study of comparable survival after neoadjuvant versus adjuvant chemotherapy in cT1-2N0M0 triple-negative breast cancer**

**Maxim Olsson^1,2†^, Slavica Janeva^3,4†^, Jari Martikainen^5^, Anna-Karin Tzikas^1,6^, Per Karlsson^1,7^, Toshima Z. Parris^1,2^**

^1^Department of Oncology, Institute of Clinical Sciences, Sahlgrenska Academy, University of Gothenburg, Gothenburg, Sweden; ^2^Sahlgrenska Center for Cancer Research, Sahlgrenska Academy, University of Gothenburg, Gothenburg, Sweden; ^3^Region Västra Götaland, Sahlgrenska University Hospital, Department of Surgery, Gothenburg, Sweden; ^4^Department of Surgery, Institute of Clinical Sciences, Sahlgrenska Academy, University of Gothenburg, Gothenburg, Sweden; ^5^Bioinformatics and Data Centre, Sahlgrenska Academy, University of Gothenburg, Gothenburg, Sweden; ^6^Region Västra Götaland, Department of Oncology, NU Hospital Group, Uddevalla, Sweden; ^7^Region Västra Götaland, Sahlgrenska University Hospital, Department of Oncology, Gothenburg, Sweden.

**Outcomes and definitions**

Patients were stratified into the AT and NACT cohorts based on administered treatment. The AT cohort only included patients who received post-operative treatment: ACT + ART (n=2254) or ACT only (n=782). As NACT is often combined with AT, the NACT cohort consisted of patients who received NACT + ACT + ART (n=178), NACT + ACT (n=49), NACT + ART (n=405), or NACT only (n=79). Premenopausal status was defined as <6 months since the last menstrual period, while postmenopausal status was defined as ≥6 months since the last menstrual period, as pre-defined by the NBCR. Using Swedish national guidelines, cases with ER and PR<10% were classified as negative, while Ki67 <20% and ≥20% were classified as low and high expression, respectively. Cases were classified as human epidermal growth factor receptor 2 (HER2)-negative for HercepTest 0, 1+ or 2+ without HER2 amplification [[1](#_ENREF_1), [2](#_ENREF_2)].

Staging was performed according to the TNM classification. Clinical stage (cT, cN, cM) was based on pre-treatment imaging and examination. Pathological stage was recorded as pT, pN, and pM after primary surgery, or ypT, ypN, and ypM following NACT. Postoperative pathological N status was based on the number of metastatic axillary lymph nodes: N0 (0), N1 (1–3), N2 (4–9), and N3 (≥10). Missing data were classified as NX.

Using in- and outpatient data, we calculated Charlson Comorbidity Indices tailored for use with Swedish registry-based data as described elsewhere [[3-5](#_ENREF_3)]. In brief, the CCI incorporates the ICD codes (revisions 7 to 10) for myocardial infarction, congestive heart failure, peripheral vascular disease, cerebrovascular disease, pulmonary diseases, rheumatic disease, dementia, hemiplegia, diabetes, chronic kidney disease, liver disease, gastric peptic ulcer disease, cancer (all malignancies including leukemia and lymphoma, but excluding breast cancer), and HIV/AIDS. CCI was then classified as weighted CCI (CCIw ranging from 0 to 10; comorbidity burden). CCIw was further stratified into CCIw 0, CCIw 1–3, and CCIw 4–10.

**Statistical analysis (Continuation)**

The survival R package (version 3.7.0) was used for underlying survival analyses, including Cox proportional hazards regression [[6](#_ENREF_6)]. Data visualizations were generated using the ggplot2 (version 3.5.1) and ggpubr (version 0.6.0) packages in R [[7](#_ENREF_7), [8](#_ENREF_8)]. The Sankey plots, which visualize transitions between baseline and post-treatment tumor T-stages, TNM-stages, and tumor subtypes, was created using the alluvial R package (version 0.1.2) [[9](#_ENREF_9)].

**References**

1. Curigliano G, Burstein HJ, Winer EP, Gnant M, Dubsky P, Loibl S, et al. De-escalating and escalating treatments for early-stage breast cancer: the St. Gallen International Expert Consensus Conference on the Primary Therapy of Early Breast Cancer 2017. Annals of Oncology. 2017;28(8):1700-12. doi: <https://doi.org/10.1093/annonc/mdx308>.

2. Jacobs TW, Gown AM, Yaziji H, Barnes MJ, Schnitt SJ. Specificity of HercepTest in Determining <i>HER-2</i>/<i>neu</i> Status of Breast Cancers Using the United States Food and Drug Administration&#x2013;Approved Scoring System. Journal of Clinical Oncology. 1999;17(7):1983-. doi: 10.1200/jco.1999.17.7.1983.

3. Ludvigsson JF AP, Askling J, Byberg L, Carrero JJ, Ekström AM, Ekström M, Smedby KE, Hagström H, James S, Järvholm B, Michaelsson K, Pedersen NL, Sundelin H, Sundquist K, Sundström J. Adaptation of the Charlson Comorbidity Index for Register-Based Research in Sweden. Clin Epidemiol. 2021;13:21-41. doi: <https://doi.org/10.2147/CLEP.S282475>.

4. Quan H, Sundararajan V, Halfon P, Fong A, Burnand B, Luthi JC, et al. Coding algorithms for defining comorbidities in ICD-9-CM and ICD-10 administrative data. Med Care. 2005;43(11):1130-9. doi: 10.1097/01.mlr.0000182534.19832.83.

5. Nyqvist-Streng J, Somi J, Martikainen J, Olsson M, Helou K, Chamalidou C, et al. Age and comorbidity in relation to treatment and survival outcomes in triple-negative breast cancer: A Swedish nationwide registry-based study. J Geriatr Oncol. 2025;16(5):102255. doi: 10.1016/j.jgo.2025.102255.

6. Therneau TM. A Package for Survival Analysis in R. 2024.

7. Wickham H. ggplot2: Elegant Graphics for Data Analysis. 2016.

8. Kassambara A. ggpubr: 'ggplot2' Based Publication Ready Plots. 2023.

9. Bojanowski M, Edwards R. alluvial: R Package for Creating Alluvial Diagrams. 2016.
