# Supplementary material for: Retrospective study of comparable survival after neoadjuvant versus adjuvant chemotherapy in cT1-2N0M0 triple-negative breast cancer
Source: Breast Cancer Res Treat. 2026 May 12;217(2):34. doi: 10.1007/s10549-026-07983-9 (PMC13167834; doi:10.1007/s10549-026-07983-9)
Supplement: Supplementary file 2 — Supplementary Material 2 [file 10549_2026_7983_MOESM2_ESM.docx]

**Supplementary Figure 1. Propensity score matching before and after matching**

Propensity score methods used to adjust for baseline differences between the NACT and AT cohorts. (**a**) Propensity score distribution plot before propensity score matching (PSM). Turquoise depicts the AT cohort, pink depicts the NACT cohort, and gray depicts the overlap, the range of propensity scores where both cohorts have observations. The x-axis shows the estimated propensity scores, derived from baseline characteristics, and used to match patients between the NACT and AT cohorts. (**b**) Propensity score distribution plot after PSM. (**c**) The jitter plot shows the distribution of the two cohorts (NACT and AT) and the results of the matching process. After excluding patients with missing data on the matching covariates (age, clinical T-stage, and comorbidities), 711 of the 711 patients in the NACT cohort remained. Thus, 711 patients in the NACT cohort were successfully matched with 711 patients in the AT cohort, leaving 2325 unmatched patients in the AT group. (**d**) Covariate balance between the NACT and AT cohorts before and after matching, shown with a Love plot. The x-axis represents the absolute standardized mean differences (SMDs) for each covariate used in the matching. Red dots indicate SMDs before matching (unadjusted), and blue dots indicate SMDs after matching (adjusted). A vertical dotted line at 0.1 marks the threshold below which covariate imbalance is considered negligible, illustrating improved balance after matching.


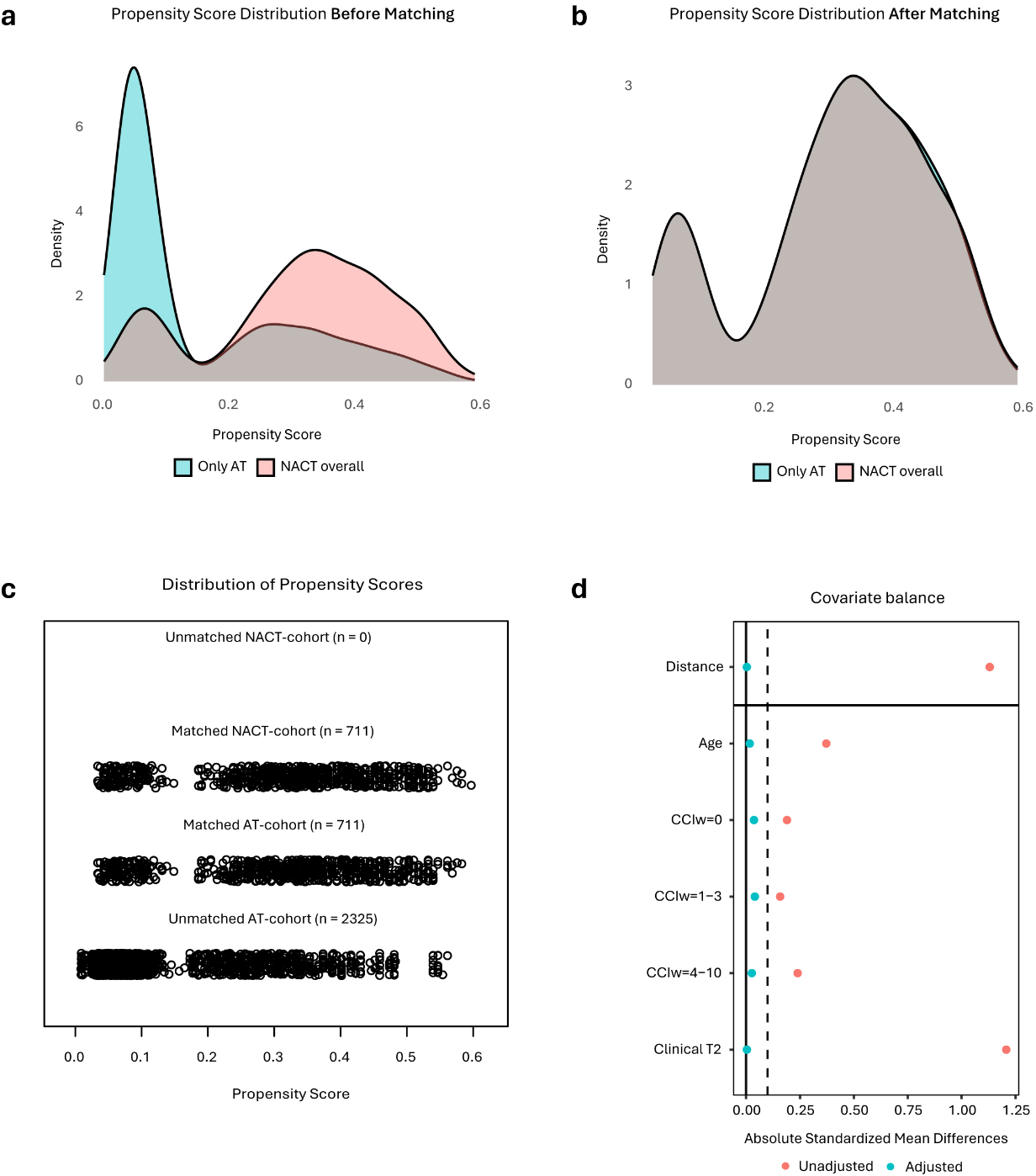


**Supplementary Figure 2. Cause of death stratified by ICD codes**

Cause of death stratified by ICD-10 codes in the (**a**) AT cohort and (**b**) NACT cohort. The y-axis lists grouped ICD-10 code categories (e.g., I00–I99: Diseases of the circulatory system), while the x-axis represents the percentage of deaths attributed to each category within the respective cohort.


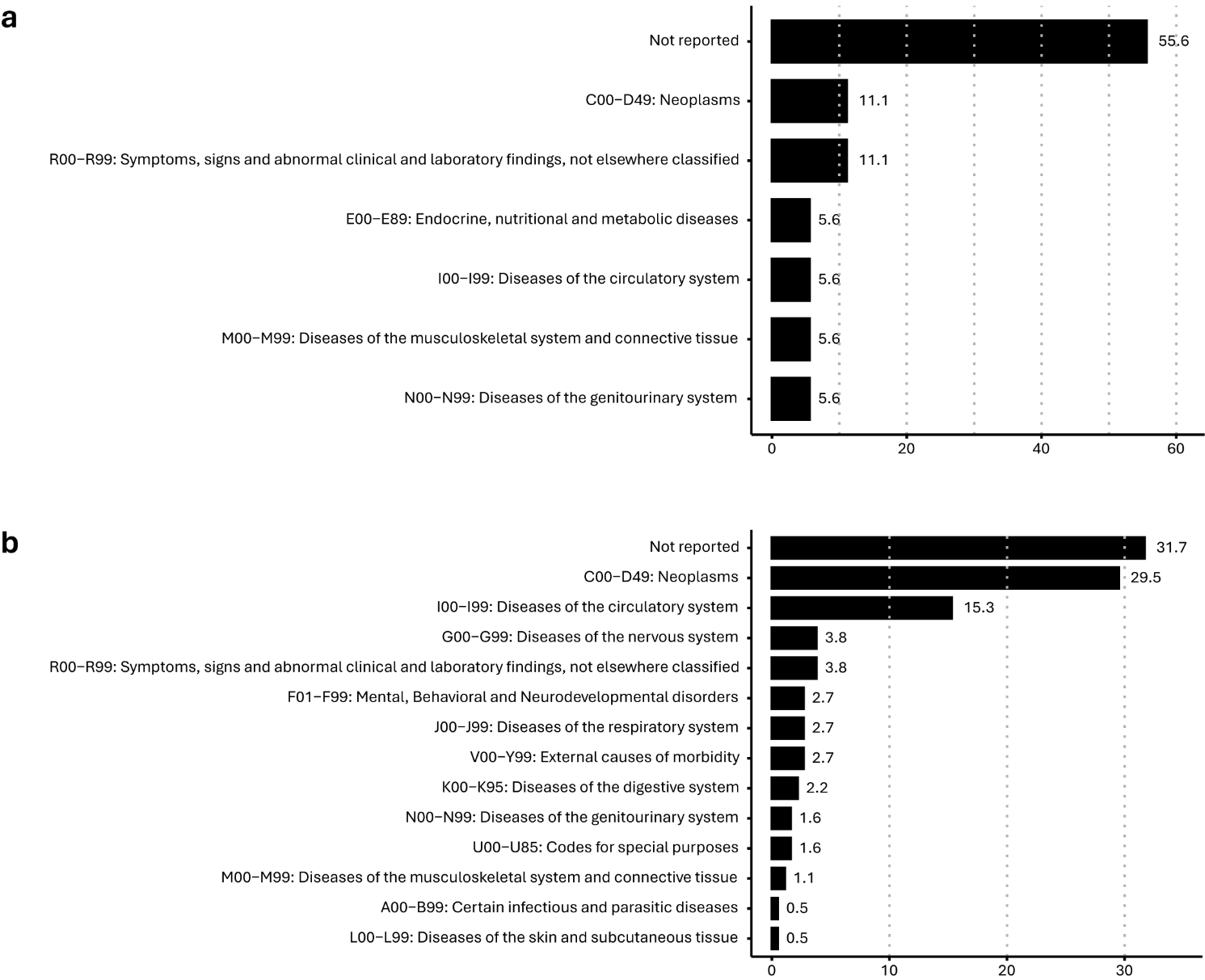


**Supplementary Figure 3. Kaplan–Meier plots on the AT cohort stratified by multimodality treatment**

Dotted vertical black lines indicate median 3- (left) and 5-year survival (right). Shaded areas represent 95% confidence intervals (95% CI). Each interval on the x-axis represents 2 years, with a total visualized follow-up time of 14 years. (**a**) Overall survival (OS) and (**b**) breast cancer-specific survival (BCSS) for the AT cohort stratified by multimodality treatment. *p < 0.05; **p ≤ 0.01; ***p ≤ 0.001; **p ≤ 0.0001.


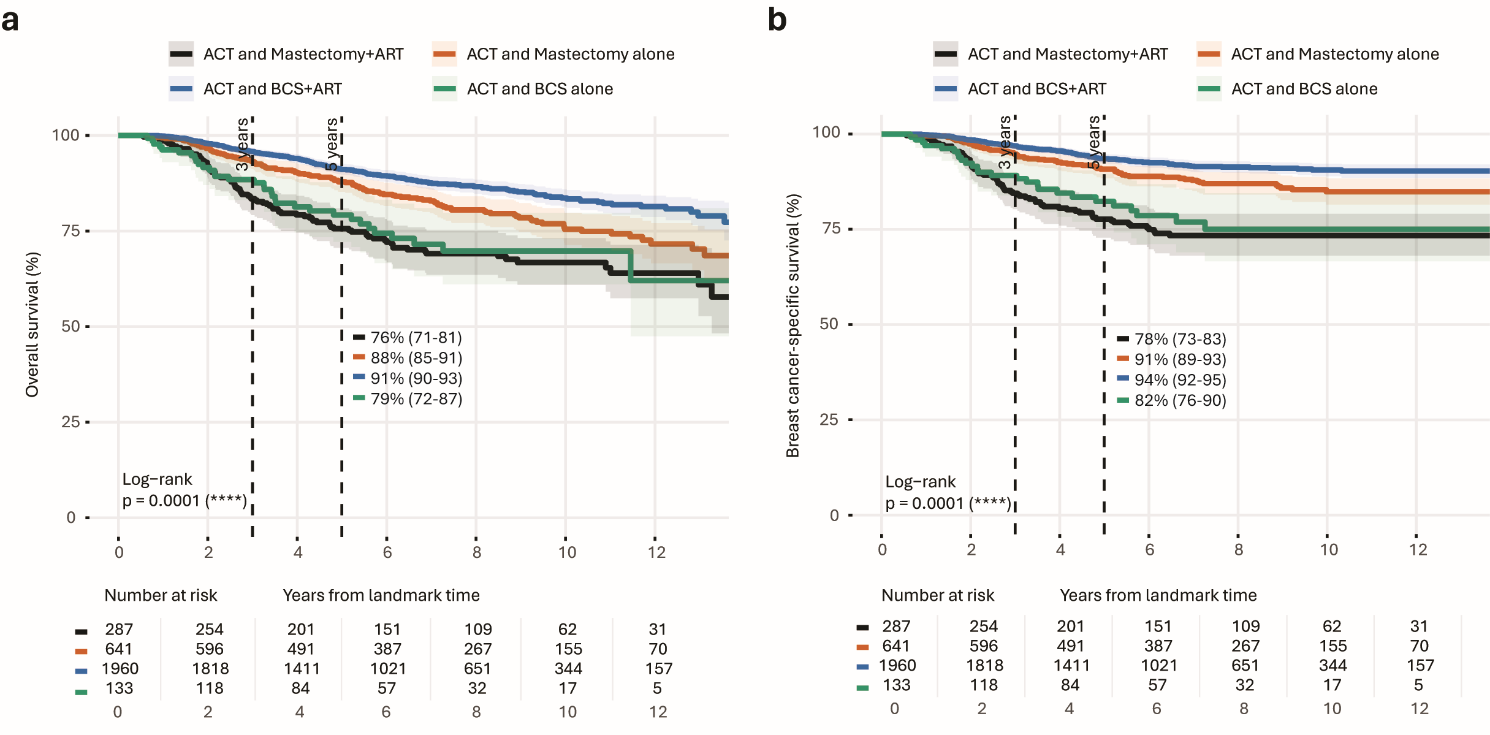


**Supplementary Figure 4. Multivariable Cox regression analysis for the AT cohort**

Forest plots showing the multivariable Cox regression analysis of factors associated with (**a**) overall survival (OS) and (**b**) breast cancer-specific survival (BCSS) in the AT cohort. Models were adjusted for multimodality treatment, patient age, tumor size, and comorbidities. Hazard ratios (HRs) with 95% confidence intervals are displayed for each variable category. Blue squares indicate the reference categories when applicable. *p < 0.05; **p ≤ 0.01; ***p ≤ 0.001; ****p ≤ 0.0001.


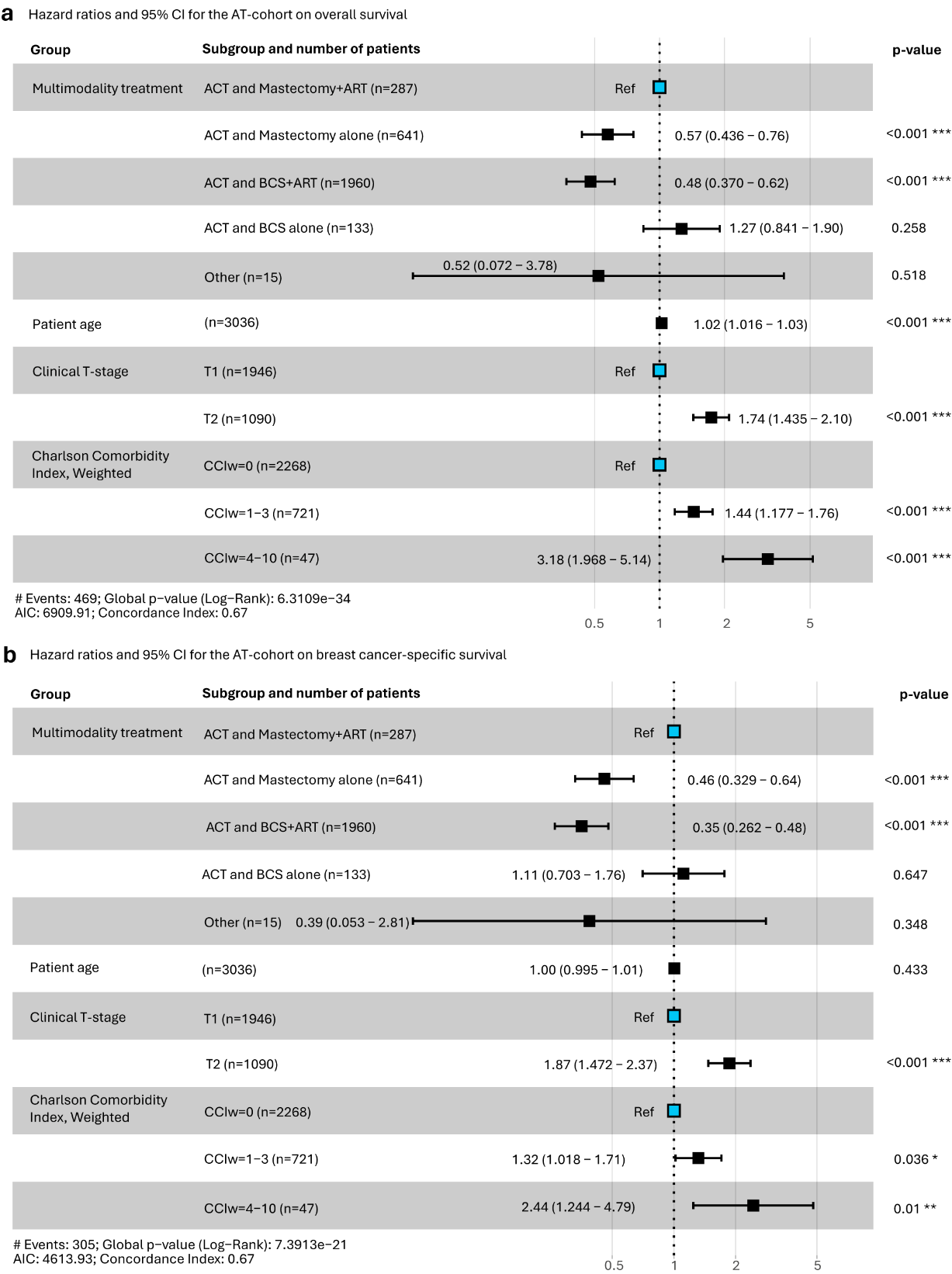

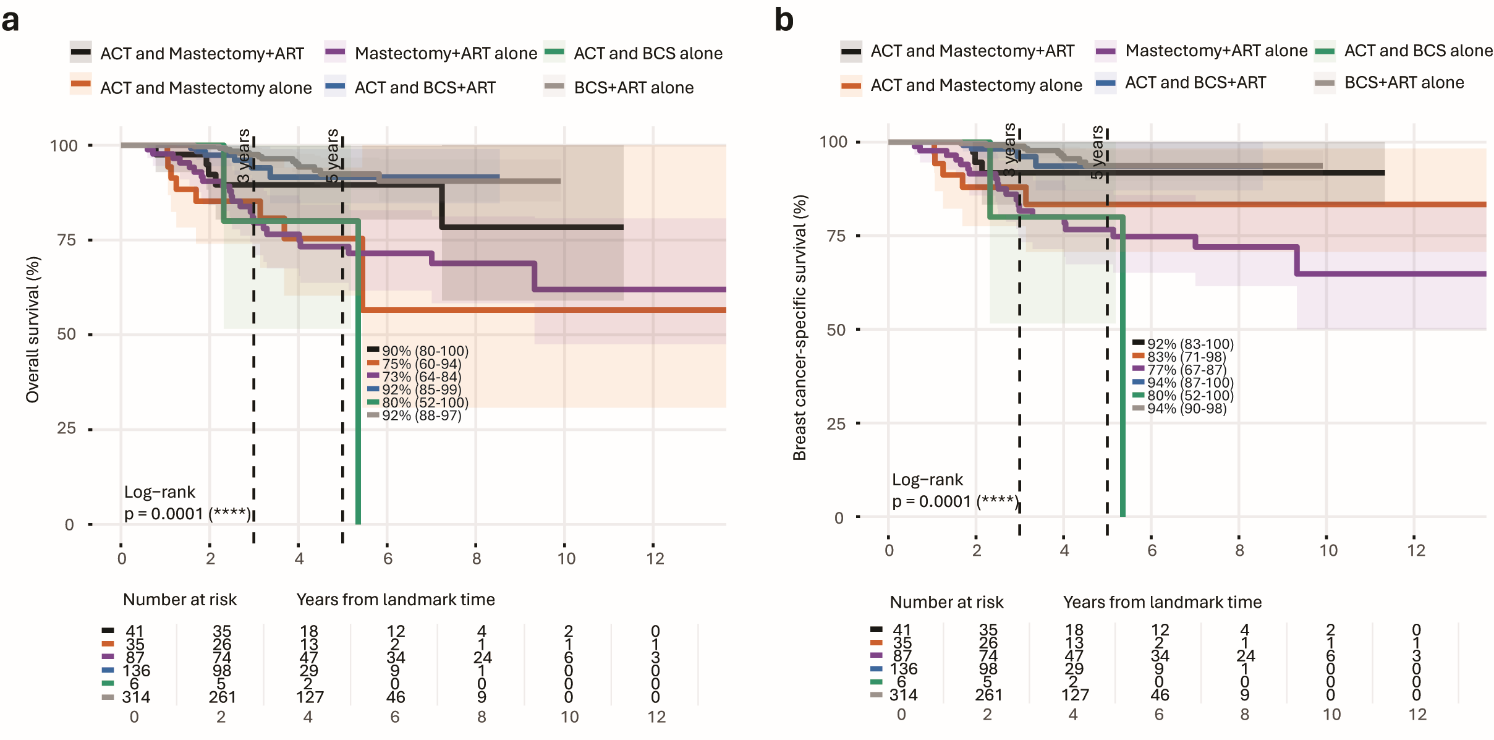


**Supplementary Figure 5. Kaplan–Meier plots on the NACT cohorts stratified by multimodality treatment**

Dotted vertical black lines indicate median 3- (left) and 5-year survival (right). Shaded areas represent 95% confidence intervals (95% CI). Each interval on the x-axis represents 2 years, with a total visualized follow-up time of 14 years. (a) Overall survival (OS) and (b) breast cancer-specific survival (BCSS) for the NACT cohort stratified by multimodality treatment. *p < 0.05; **p ≤ 0.01; ***p ≤ 0.001; **p ≤ 0.0001.

**Supplementary Figure 6. Multivariable Cox regression analysis for the NACT cohort**

Forest plots showing the multivariable Cox regression analysis of factors associated with (**a**) overall survival (OS) and (**b**) breast cancer-specific survival (BCSS) in the NACT cohort. Models were adjusted for multimodality treatment, patient age, tumor size, and comorbidities. Hazard ratios (HRs) with 95% confidence intervals are displayed for each variable category. Blue squares indicate the reference categories when applicable. *p < 0.05; **p ≤ 0.01; ***p ≤ 0.001; ****p ≤ 0.0001.


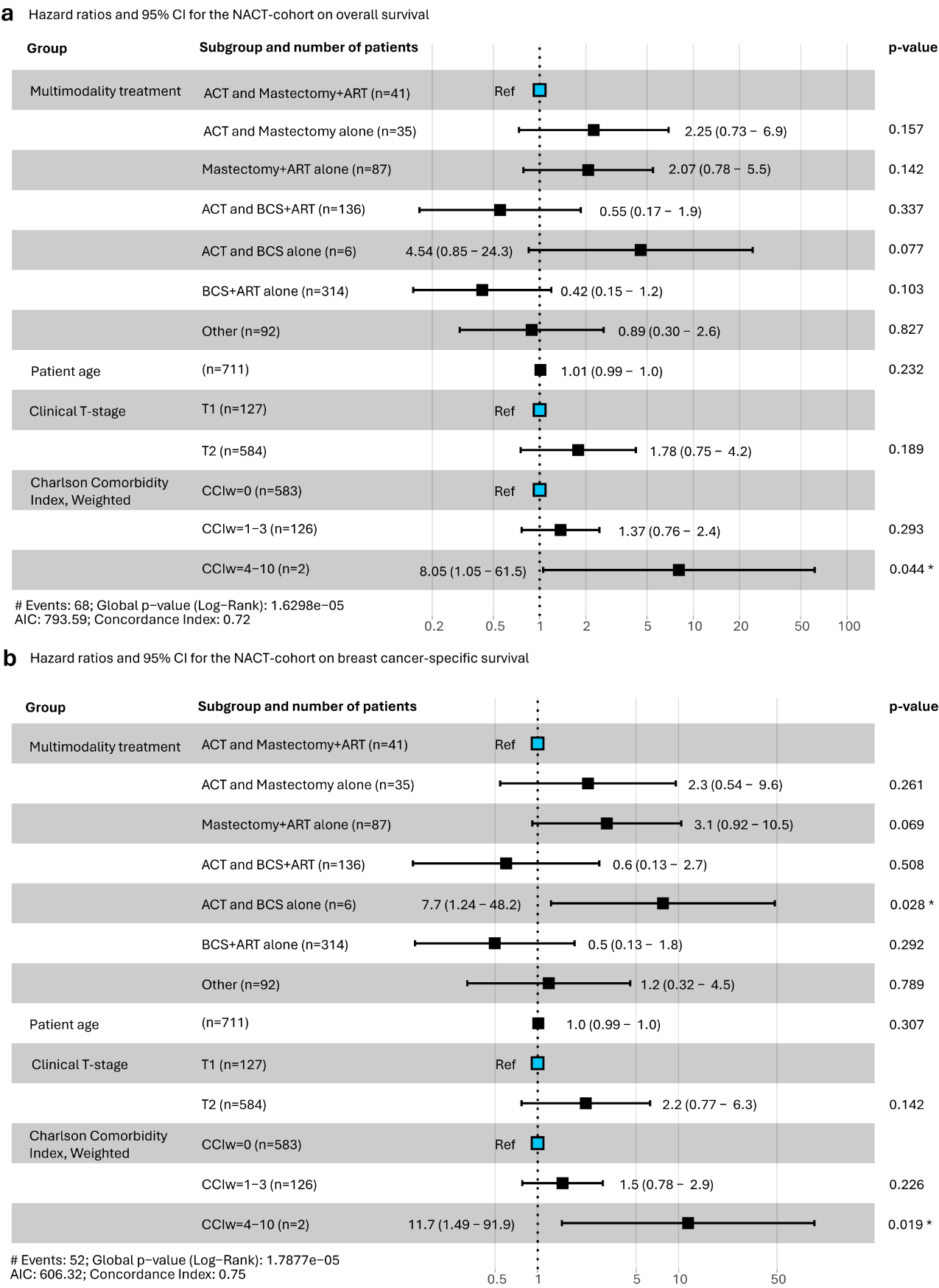


**Supplementary Table 1.** Clinicopathological characteristics and treatment details of 3036 TNBC patients in the AT cohort, stratified by adjuvant treatment, before and after PSM

|  | AT-cohort data before PSM |  |  |  |  | AT-cohort data after PSM |  |  |  |
| --- | --- | --- | --- | --- | --- | --- | --- | --- | --- |
| Characteristics | Overall (n=3036) | ACT and ART (n=2254;74%) | ACT alone (n=782;26%) | Global p-value |  | Overall (n=711) | ACT and ART (n=476;16%) | ACT alone (n=235;8%) | Global p-value |
| **Patient age at baseline, years (median [IQR])** | **59.00 [48.00,67.00]** | **58.00 [49.00,67.00]** | **60.00 [46.00,69.00]** | **0.386** |  | **53.00 [43.00,62.00]** | **53.00 [43.75,62.00]** | **53.00 [41.00,64.00]** | **0.826** |
| Tumor size (mm) at baseline (median [IQR]) | 16.00 [12.00,31.00] | 13.00 [10.00,27.00] | 26.50 [24.25,28.75] | 0.38 |  | 31.00 [26.50,34.50] | 38.00 [38.00,38.00] | 26.50 [24.25,28.75] | 0.221 |
| Tumor size (mm) post-surgery (median [IQR]) | 19.00 [13.50,25.00] | 18.00 [13.00,25.00] | 20.00 [15.00,28.00] | <0.001 |  | 25.00 [19.00,31.00] | 24.00 [19.00,31.00] | 25.00 [19.00,32.00] | 0.618 |
| Ki67% at baseline (median [IQR]) | 40.00 [30.00,75.00] | 62.50 [42.50,78.75] | 30.00 [26.00,35.00] | 0.154 |  | 40.00 [22.00,40.00] | 40.00 [24.50,57.50] | 31.00 [26.50,35.50] | 0.767 |
| Ki67% post-surgery (median [IQR]) | 68.00 [42.00,82.00] | 67.00 [42.00,80.00] | 70.00 [46.00,85.00] | 0.016 |  | 70.00 [46.00,85.00] | 70.00 [46.00,83.00] | 71.00 [47.50,85.00] | 0.473 |
| Swedish healthcare region, n (%) |  |  |  |  |  |  |  |  |  |
| Mid Sweden | 597 (19.7) | 452 (20.1) | 145 (18.5) | 0.002 |  | 137 (19.3) | 99 (20.8) | 38 (16.2) | 0.026 |
| North | 272 (9.0) | 211 (9.4) | 61 (7.8) |  |  | 42 (5.9) | 30 (6.3) | 12 (5.1) |  |
| South | 512 (16.9) | 372 (16.5) | 140 (17.9) |  |  | 142 (20.0) | 90 (18.9) | 52 (22.1) |  |
| Southeast | 336 (11.1) | 239 (10.6) | 97 (12.4) |  |  | 110 (15.5) | 75 (15.8) | 35 (14.9) |  |
| Stockholm/Gotland | 551 (18.1) | 439 (19.5) | 112 (14.3) |  |  | 154 (21.7) | 112 (23.5) | 42 (17.9) |  |
| West | 768 (25.3) | 541 (24.0) | 227 (29.0) |  |  | 126 (17.7) | 70 (14.7) | 56 (23.8) |  |
| Age range at diagnosis, years, n (%) |  |  |  |  |  |  |  |  |  |
| <40 | 307 (10.1) | 193 (8.6) | 114 (14.6) | <0.001 |  | 126 (17.7) | 74 (15.5) | 52 (22.1) | 0.054 |
| 40-49 | 533 (17.6) | 410 (18.2) | 123 (15.7) |  |  | 171 (24.1) | 124 (26.1) | 47 (20.0) |  |
| 50-64 | 1178 (38.8) | 923 (40.9) | 255 (32.6) |  |  | 271 (38.1) | 187 (39.3) | 84 (35.7) |  |
| 65-74 | 826 (27.2) | 619 (27.5) | 207 (26.5) |  |  | 113 (15.9) | 75 (15.8) | 38 (16.2) |  |
| >=75 | 192 (6.3) | 109 (4.8) | 83 (10.6) |  |  | 30 (4.2) | 16 (3.4) | 14 (6.0) |  |
| Year of diagnosis (%) |  |  |  |  |  |  |  |  |  |
| 2007-2008 | 56 (1.8) | 39 (1.7) | 17 (2.2) | <0.001 |  | 13 (1.8) | 8 (1.7) | 5 (2.1) | 0.055 |
| 2009-2010 | 295 (9.7) | 206 (9.1) | 89 (11.4) |  |  | 76 (10.7) | 45 (9.5) | 31 (13.2) |  |
| 2011-2012 | 411 (13.5) | 277 (12.3) | 134 (17.1) |  |  | 106 (14.9) | 64 (13.4) | 42 (17.9) |  |
| 2013-2014 | 565 (18.6) | 417 (18.5) | 148 (18.9) |  |  | 142 (20.0) | 95 (20.0) | 47 (20.0) |  |
| 2015-2016 | 576 (19.0) | 422 (18.7) | 154 (19.7) |  |  | 131 (18.4) | 84 (17.6) | 47 (20.0) |  |
| 2017-2018 | 512 (16.9) | 402 (17.8) | 110 (14.1) |  |  | 125 (17.6) | 89 (18.7) | 36 (15.3) |  |
| 2019-2020 | 478 (15.7) | 378 (16.8) | 100 (12.8) |  |  | 100 (14.1) | 80 (16.8) | 20 (8.5) |  |
| 2021 | 143 (4.7) | 113 (5.0) | 30 (3.8) |  |  | 18 (2.5) | 11 (2.3) | 7 (3.0) |  |
| Menopausal status at baseline (%) |  |  |  |  |  |  |  |  |  |
| Premenopausal | 836 (27.5) | 604 (26.8) | 232 (29.7) | 0.255 |  | 285 (40.1) | 190 (39.9) | 95 (40.4) | 0.555 |
| Postmenopausal | 1955 (64.4) | 1470 (65.2) | 485 (62.0) |  |  | 367 (51.6) | 250 (52.5) | 117 (49.8) |  |
| Missing data | 245 (8.1) | 180 (8.0) | 65 (8.3) |  |  | 59 (8.3) | 36 (7.6) | 23 (9.8) |  |
| **Clinical T stage (cT; %)** |  |  |  |  |  |  |  |  |  |
| T1 | 1946 (64.1) | 1551 (68.8) | 395 (50.5) | <0.001 |  | 126 (17.7) | 92 (19.3) | 34 (14.5) | 0.136 |
| T2 | 1090 (35.9) | 703 (31.2) | 387 (49.5) |  |  | 585 (82.3) | 384 (80.7) | 201 (85.5) |  |
| Clinical stage (cTNM)(%) |  |  |  |  |  |  |  |  |  |
| IA | 1946 (64.1) | 1551 ( 68.8) | 395 ( 50.5) | <0.001 |  | 126 (17.7) | 92 (19.3) | 34 (14.5) | 0.136 |
| IIA | 1090 (35.9) | 703 ( 31.2) | 387 ( 49.5) |  |  | 585 (82.3) | 384 (80.7) | 201 (85.5) |  |
| Pathological subtype (%) |  |  |  |  |  |  |  |  |  |
| TNBC | 3036 (100) | 2254 (100.0) | 782 (100.0) | NA |  | 711 (100.0) | 2254 (100.0) | 782 (100.0) | NA |
| Luminal A | 0 (0.0) | 0 (0.0) | 0 (0.0) |  |  | 0 (0.0) | 0 (0.0) | 0 (0.0) |  |
| Luminal B/HER2- | 0 (0.0) | 0 (0.0) | 0 (0.0) |  |  | 0 (0.0) | 0 (0.0) | 0 (0.0) |  |
| Non-luminal HER2+ | 0 (0.0) | 0 (0.0) | 0 (0.0) |  |  | 0 (0.0) | 0 (0.0) | 0 (0.0) |  |
| Unspecified (ER-,PR+,HER2-) | 0 (0.0) | 0 (0.0) | 0 (0.0) |  |  | 0 (0.0) | 0 (0.0) | 0 (0.0) |  |
| Pathological T stage (ypT; %) |  |  |  |  |  |  |  |  |  |
| T0 | 1 (0.0) | 1 (0.0) | 0 (0.0) | <0.001 |  | 0 (0.0) | 0 (0.0) | 0 (0.0) | 0.131 |
| T1 | 1796 (59.2) | 1402 (62.2) | 394 (50.4) |  |  | 237 (33.3) | 163 (34.2) | 74 (31.5) |  |
| T2 | 1182 (38.9) | 800 (35.5) | 382 (48.8) |  |  | 455 (64.0) | 296 (62.2) | 159 (67.7) |  |
| T3 | 44 (1.4) | 39 (1.7) | 5 (0.6) |  |  | 16 (2.3) | 14 (2.9) | 2 (0.9) |  |
| Missing data | 13 (0.4) | 12 (0.5) | 1 (0.1) |  |  | 3 (0.4) | 3 (0.6) | 0 (0.0) |  |
| Pathological N stage (ypN; %) |  |  |  |  |  |  |  |  |  |
| N0 | 2391 (78.8) | 1689 (74.9) | 702 (89.8) | <0.001 |  | 531 (74.7) | 325 (68.3) | 206 (87.7) | <0.001 |
| N1 | 525 (17.3) | 461 (20.5) | 64 (8.2) |  |  | 148 (20.8) | 126 (26.5) | 22 (9.4) |  |
| N2 | 48 (1.6) | 46 (2.0) | 2 (0.3) |  |  | 15 (2.1) | 15 (3.2) | 0 (0.0) |  |
| N3 | 25 (0.8) | 25 (1.1) | 0 (0.0) |  |  | 6 (0.8) | 6 (1.3) | 0 (0.0) |  |
| NX | 47 (1.5) | 33 (1.5) | 14 (1.8) |  |  | 11 (1.5) | 4 (0.8) | 7 (3.0) |  |
| Pathological stage (ypTNM)(%) |  |  |  |  |  |  |  |  |  |
| IA | 1517 (50.0) | 1154 (51.2) | 363 (46.4) | <0.001 |  | 192 (27.0) | 127 (26.7) | 65 (27.7) | <0.001 |
| IIA | 1080 (35.6) | 723 (32.1) | 357 (45.7) |  |  | 371 (52.2) | 226 (47.5) | 145 (61.7) |  |
| IIB | 290 (9.6) | 245 (10.9) | 45 (5.8) |  |  | 106 (14.9) | 88 (18.5) | 18 (7.7) |  |
| IIIA | 65 (2.1) | 63 (2.8) | 2 (0.3) |  |  | 22 (3.1) | 22 (4.6) | 0 (0.0) |  |
| IIIC | 25 (0.8) | 25 (1.1) | 0 (0.0) |  |  | 6 (0.8) | 6 (1.3) | 0 (0.0) |  |
| Unspecified | 59 (1.9) | 44 (2.0) | 15 (1.9) |  |  | 14 (2.0) | 7 (1.5) | 7 (3.0) |  |
| NHG (%) |  |  |  |  |  |  |  |  |  |
| Grade1 | 27 (0.9) | 21 (0.9) | 6 (0.8) | 0.225 |  | 5 (0.7) | 4 (0.8) | 1 (0.4) | 0.863 |
| Grade2 | 434 (14.3) | 339 (15.0) | 95 (12.1) |  |  | 82 (11.5) | 57 (12.0) | 25 (10.6) |  |
| Grade3 | 2537 (83.6) | 1865 (82.7) | 672 (85.9) |  |  | 616 (86.6) | 410 (86.1) | 206 (87.7) |  |
| Missing data | 38 (1.3) | 29 (1.3) | 9 (1.2) |  |  | 8 (1.1) | 5 (1.1) | 3 (1.3) |  |
| Survival status (%) |  |  |  |  |  |  |  |  |  |
| Alive | 2567 (84.6) | 1942 (86.2) | 625 (79.9) | <0.001 |  | 586 (82.4) | 392 (82.4) | 194 (82.6) | 0.642 |
| Death by BC | 286 (9.4) | 189 (8.4) | 97 (12.4) |  |  | 92 (12.9) | 64 (13.4) | 28 (11.9) |  |
| Death by other causes | 183 (6.0) | 123 (5.5) | 60 (7.7) |  |  | 33 (4.6) | 20 (4.2) | 13 (5.5) |  |
| **Charlson comorbidity index, weighted, grouped (%)** |  |  |  |  |  |  |  |  |  |
| CCIw=0 | 2268 (74.7) | 1745 (77.4) | 523 (66.9) | <0.001 |  | 593 (83.4) | 409 (85.9) | 184 (78.3) | 0.025 |
| CCIw=1-3 | 721 (23.7) | 478 (21.2) | 243 (31.1) |  |  | 115 (16.2) | 66 (13.9) | 49 (20.9) |  |
| CCIw=4-10 | 47 (1.5) | 31 (1.4) | 16 (2.0) |  |  | 3 (0.4) | 1 (0.2) | 2 (0.9) |  |
| Surgery (%) |  |  |  |  |  |  |  |  |  |
| Mastectomy | 928 (30.6) | 287 (12.7) | 641 (82.0) | <0.001 |  | 300 (42.2) | 98 (20.6) | 202 (86.0) | <0.001 |
| Breast-conserving surgery | 2093 (68.9) | 1960 (87.0) | 133 (17.0) |  |  | 404 (56.8) | 375 (78.8) | 29 (12.3) |  |
| Subcutaneous mastectomy | 11 (0.4) | 3 (0.1) | 8 (1.0) |  |  | 5 (0.7) | 1 (0.2) | 4 (1.7) |  |
| Only axilla surgery | 1 (0.0) | 1 (0.0) | 0 (0.0) |  |  | 0 (0.0) | 0 (0.0) | 0 (0.0) |  |
| Missing data | 3 (0.1) | 3 (0.1) | 0 (0.0) |  |  | 2 (0.3) | 2 (0.4) | 0 (0.0) |  |
| Axillary surgery (%) |  |  |  |  |  |  |  |  |  |
| SN | 2494 (82.1) | 1810 (80.3) | 684 (87.5) | <0.001 |  | 536 (75.4) | 339 (71.2) | 197 (83.8) | <0.001 |
| ALND | 116 (3.8) | 87 (3.9) | 29 (3.7) |  |  | 44 (6.2) | 33 (6.9) | 11 (4.7) |  |
| SN and ALND | 366 (12.1) | 319 (14.2) | 47 (6.0) |  |  | 119 (16.7) | 100 (21.0) | 19 (8.1) |  |
| Sampling | 45 (1.5) | 28 (1.2) | 17 (2.2) |  |  | 9 (1.3) | 3 (0.6) | 6 (2.6) |  |
| Missing data | 15 (0.5) | 10 (0.4) | 5 (0.6) |  |  | 3 (0.4) | 1 (0.2) | 2 (0.9) |  |
| Neoadjuvant chemotherapy (%) |  |  |  |  |  |  |  |  |  |
| Yes | 0 (0.0) | 0 (0.0) | 0 (0.0) | NA |  | 0 (0.0) | 0 (0.0) | 0 (0.0) | NA |
| No | 3036 (100.0) | 2254 (100.0) | 782 (100.0) |  |  | 711 (100.0) | 476 (100.0) | 235 (100.0) |  |
| Completed NACT according to plan (%) |  |  |  |  |  |  |  |  |  |
| Yes | 0 (0.0) | 0 (0.0) | 0 (0.0) | NA |  | 0 (0.0) | 0 (0.0) | 0 (0.0) | NA |
| No | 0 (0.0) | 0 (0.0) | 0 (0.0) |  |  | 0 (0.0) | 0 (0.0) | 0 (0.0) |  |
| Missing data | 3036 (100.0) | 2254 (100.0) | 782 (0.0) |  |  | 711 (100.0) | 476 (100.0) | 235 (100.0) |  |
| Reason for discontinuation of NACT (%) |  |  |  |  |  |  |  |  |  |
| Side effects | 0 (0.0) | 0 (0.0) | 0 (0.0) | NA |  | 0 (0.0) | 0 (0.0) | 0 (0.0) | NA |
| Other | 0 (0.0) | 0 (0.0) | 0 (0.0) |  |  | 0 (0.0) | 0 (0.0) | 0 (0.0) |  |
| Missing data | 3036 (100.0) | 2254 (100.0) | 782 (0.0) |  |  | 711 (100.0) | 476 (100.0) | 235 (100.0) |  |
| NACT administered (%) |  |  |  |  |  |  |  |  |  |
| Anthracycline-based | 0 (0.0) | 0 (0.0) | 0 (0.0) | NA |  | 0 (0.0) | 0 (0.0) | 0 (0.0) | NA |
| Anthracycline+taxane | 0 (0.0) | 0 (0.0) | 0 (0.0) |  |  | 0 (0.0) | 0 (0.0) | 0 (0.0) |  |
| Taxane-based | 0 (0.0) | 0 (0.0) | 0 (0.0) |  |  | 0 (0.0) | 0 (0.0) | 0 (0.0) |  |
| Unknown | 0 (0.0) | 0 (0.0) | 0 (0.0) |  |  | 0 (0.0) | 0 (0.0) | 0 (0.0) |  |
| None | 3036 (100.0) | 2254 (100.0) | 782 (0.0) |  |  | 711 (0.0) | 476 (100.0) | 235 (100.0) |  |
| Adjuvant chemotherapy (%) |  |  |  |  |  |  |  |  |  |
| Yes | 3036 (100.0) | 2254 (100.0) | 782 (100.0) | NA |  | 711 (100.0) | 476 (100.0) | 235 (100.0) | NA |
| No | 0 (0.0) | 0 (0.0) | 0 (0.0) |  |  | 0 (0.0) | 0 (0.0) | 0 (0.0) |  |
| Completed ACT according to plan (%) |  |  |  |  |  |  |  |  |  |
| Yes | 2351 (77.4) | 1764 (78.3) | 587 (75.1) | 0.137 |  | 551 (77.5) | 375 (78.8) | 176 (74.9) | 0.265 |
| No | 666 (21.9) | 475 (21.1) | 191 (24.4) |  |  | 152 (21.4) | 94 (19.7) | 58 (24.7) |  |
| Missing data | 19 (0.7) | 15 (0.6) | 4 (0.5) |  |  | 8 (1.1) | 7 (1.4) | 1 (0.4) |  |
| Reason for discontinuation of ACT (%) |  |  |  |  |  |  |  |  |  |
| Side effects | 566 (18.6) | 410 (18.2) | 156 (19.9) | 0.011 |  | 128 (18.0) | 87 (18.3) | 41 (17.4) | <0.001 |
| Other | 93 (3.1) | 58 (2.6) | 35 (4.5) |  |  | 22 (3.1) | 5 (1.1) | 17 (7.2) |  |
| Missing data | 2377 (78.3) | 1786 (79.2) | 591 (75.6) |  |  | 561 (78.9) | 384 (80.7) | 177 (75.3) |  |
| ACT administered (%) |  |  |  |  |  |  |  |  |  |
| Anthracycline-based | 1183 (39.0) | 862 (38.2) | 321 (41.0) | <0.001 |  | 291 (40.9) | 187 (39.3) | 104 (44.3) | <0.001 |
| Anthracycline+taxane | 1580 (52.0) | 1226 (54.4) | 354 (45.3) |  |  | 369 (51.9) | 268 (56.3) | 101 (43.0) |  |
| Taxane-based | 149 (4.9) | 96 (4.3) | 53 (6.8) |  |  | 23 (3.2) | 9 (1.9) | 14 (6.0) |  |
| Other | 124 (4.1) | 70 (3.1) | 54 (6.9) |  |  | 28 (3.9) | 12 (2.5) | 16 (6.8) |  |
| None | 0 (0.0) | 0 (0.0) | 0 (0.0) |  |  | 0 (0.0) | 0 (0.0) | 0 (0.0) |  |
| Adjuvant radiotherapy (%) |  |  |  |  |  |  |  |  |  |
| Yes | 2254 (74.2) | 2254 (100.0) | 0 (0.0) | <0.001 |  | 476 (66.9) | 476 (100.0) | 0 (0.0) | <0.001 |
| No | 782 (25.8) | 0 (0.0) | 782 (100.0) |  |  | 235 (33.1) | 0 (0.0) | 235 (100.0) |  |
| Locoregional treatment (%) |  |  |  |  |  |  |  |  |  |
| BCS + ART | 1960 (64.6) | 1960 (87.0) | 0 (0.0) | <0.001 |  | 375 (52.7) | 375 (78.8) | 0 (0.0) | <0.001 |
| Mastectomy only | 641 (21.1) | 0 (0.0) | 641 (82.0) |  |  | 202 (28.4) | 0 (0.0) | 202 (86.0) |  |
| Masectomy + ART | 287 (9.5) | 287 (12.7) | 0 (0.0) |  |  | 98 (13.8) | 98 (20.6) | 0 (0.0) |  |
| Other | 148 (4.9) | 7 (0.3) | 141 (18.0) |  |  | 36 (5.1) | 3 (0.6) | 33 (14.0) |  |
| P-values were calculated using Chi-square test for categorical variables (with continuity correction) and ANOVA for continuous variables. Variables in bold were included in the propensity score matching. Abbreviations: ACT = Adjuvant chemotherapy; ALND = Axillary lymph node dissection; ANOVA = Analysis of variance; ART = Adjuvant radiotherapy; AT = Adjuvant therapy; BC = Breast cancer; BCS = Breast-conserving surgery; CCIw = Charlson Comorbidity Index, weighted; ER = Estrogen receptor; HER2 = Human epidermal growth factor receptor2; IQR = Interquartile range; NA = Not available; NACT = Neoadjuvant chemotherapy; NaN = Not a number; NHG = Nottingham histologic grade; NX = Nodal status unknown; PR = Progesterone receptor; PSM = Propensity score matching; SN = Sentinel node; TNBC = Triple-negative breast cancer. | | | | | | | | | |

**Supplementary Table 2.** Clinicopathological characteristics and treatment details of 711 TNBC patients in the NACT cohort, stratified by adjuvant treatment, before and after PSM

|  | NACT-cohort data before PSM |  |  |  |  |  |  | NACT-cohort data after PSM |  |  |  |  |  |
| --- | --- | --- | --- | --- | --- | --- | --- | --- | --- | --- | --- | --- | --- |
| Characteristics | Overall (n=711) | NACT+ACT+ART (n=178;25%) | NACT+ACT (n=49;7%) | NACT+ART (n=405;60%) | NACT alone (n=79;11%) | p-value |  | Overall (n=711) | NACT+ACT+ART (n=178;25%) | NACT+ACT (n=49;7%) | NACT+ART (n=405;60%) | NACT alone (n=79;11%) | p-value |
| **Patient age at baseline, years (median [IQR])** | **53.00 [43.00,62.00]** | **54.50 [46.00,62.00]** | **49.00 [43.00,62.00]** | **54.00 [42.00,62.00]** | **50.00 [41.00,64.00]** | **0.726** |  | **53.00 [43.00,62.00]** | **54.50 [46.00,62.00]** | **49.00 [43.00,62.00]** | **54.00 [42.00,62.00]** | **50.00 [41.00,64.00]** | **0.726** |
| Tumor size (mm) at baseline (median [IQR]) | 27.00 [22.00,35.00] | 28.00 [22.00,36.00] | 33.00 [23.50,40.00] | 26.00 [23.00,35.00] | 25.00 [20.50,30.00] | 0.429 |  | 27.00 [22.00,35.00] | 28.00 [22.00,36.00] | 33.00 [23.50,40.00] | 26.00 [23.00,35.00] | 25.00 [20.50,30.00] | 0.429 |
| Tumor size (mm) post-surgery (median [IQR]) | 13.00 [6.00,21.00] | 15.00 [9.00,22.00] | 15.00 [6.00,24.75] | 10.00 [4.00,20.00] | 10.00 [2.75,20.00] | 0.001 |  | 13.00 [6.00,21.00] | 15.00 [9.00,22.00] | 15.00 [6.00,24.75] | 10.00 [4.00,20.00] | 10.00 [2.75,20.00] | 0.001 |
| Ki67% at baseline (median [IQR]) | 70.00 [49.00,83.00] | 69.00 [43.00,85.00] | 65.00 [47.75,89.00] | 70.00 [50.00,80.00] | 75.00 [56.00,86.25] | 0.241 |  | 70.00 [49.00,83.00] | 69.00 [43.00,85.00] | 65.00 [47.75,89.00] | 70.00 [50.00,80.00] | 75.00 [56.00,86.25] | 0.241 |
| Ki67% post-surgery (median [IQR]) | 47.00 [12.00,80.00] | 46.00 [15.00,80.00] | 62.00 [30.50,85.00] | 42.00 [10.00,75.00] | 37.50 [14.25,75.00] | 0.065 |  | 47.00 [12.00,80.00] | 46.00 [15.00,80.00] | 62.00 [30.50,85.00] | 42.00 [10.00,75.00] | 37.50 [14.25,75.00] | 0.065 |
| Swedish healthcare region, n (%) |  |  |  |  |  |  |  |  |  |  |  |  |  |
| Mid Sweden | 63 (8.9) | 12 (6.7) | 1 (2.0) | 44 (10.9) | 6 (7.6) | <0.001 |  | 63 (8.9) | 12 (6.7) | 1 (2.0) | 44 (10.9) | 6 (7.6) | <0.001 |
| North | 26 (3.7) | 10 (5.6) | 5 (10.2) | 9 (2.2) | 2 (2.5) |  |  | 26 (3.7) | 10 (5.6) | 5 (10.2) | 9 (2.2) | 2 (2.5) |  |
| South | 193 (27.1) | 23 (12.9) | 7 (14.3) | 122 (30.1) | 41 (51.9) |  |  | 193 (27.1) | 23 (12.9) | 7 (14.3) | 122 (30.1) | 41 (51.9) |  |
| Southeast | 80 (11.3) | 26 (14.6) | 13 (26.5) | 34 (8.4) | 7 (8.9) |  |  | 80 (11.3) | 26 (14.6) | 13 (26.5) | 34 (8.4) | 7 (8.9) |  |
| Stockholm/Gotland | 255 (35.9) | 76 (42.7) | 14 (28.6) | 145 (35.8) | 20 (25.3) |  |  | 255 (35.9) | 76 (42.7) | 14 (28.6) | 145 (35.8) | 20 (25.3) |  |
| West | 94 (13.2) | 31 (17.4) | 9 (18.4) | 51 (12.6) | 3 (3.8) |  |  | 94 (13.2) | 31 (17.4) | 9 (18.4) | 51 (12.6) | 3 (3.8) |  |
| Age range at diagnosis, years, n (%) |  |  |  |  |  |  |  |  |  |  |  |  |  |
| <40 | 124 (17.4) | 27 (15.2) | 7 (14.3) | 74 (18.3) | 16 (20.3) | 0.219 |  | 124 (17.4) | 27 (15.2) | 7 (14.3) | 74 (18.3) | 16 (20.3) | 0.219 |
| 40-49 | 178 (25.0) | 41 (23.0) | 19 (38.8) | 96 (23.7) | 22 (27.8) |  |  | 178 (25.0) | 41 (23.0) | 19 (38.8) | 96 (23.7) | 22 (27.8) |  |
| 50-64 | 266 (37.4) | 76 (42.7) | 14 (28.6) | 155 (38.3) | 21 (26.6) |  |  | 266 (37.4) | 76 (42.7) | 14 (28.6) | 155 (38.3) | 21 (26.6) |  |
| 65-74 | 115 (16.2) | 29 (16.3) | 6 (12.2) | 66 (16.3) | 14 (17.7) |  |  | 115 (16.2) | 29 (16.3) | 6 (12.2) | 66 (16.3) | 14 (17.7) |  |
| >=75 | 28 (3.9) | 5 (2.8) | 3 (6.1) | 14 (3.5) | 6 (7.6) |  |  | 28 (3.9) | 5 (2.8) | 3 (6.1) | 14 (3.5) | 6 (7.6) |  |
| Year of diagnosis (%) |  |  |  |  |  |  |  |  |  |  |  |  |  |
| 2007-2008 | 3 (0.4) | 0 (0.0) | 0 (0.0) | 3 (0.7) | 0 (0.0) | <0.001 |  | 3 (0.4) | 0 (0.0) | 0 (0.0) | 3 (0.7) | 0 (0.0) | <0.001 |
| 2009-2010 | 5 (0.7) | 0 (0.0) | 1 (2.0) | 4 (1.0) | 0 (0.0) |  |  | 5 (0.7) | 0 (0.0) | 1 (2.0) | 4 (1.0) | 0 (0.0) |  |
| 2011-2012 | 8 (1.1) | 3 (1.7) | 0 (0.0) | 5 (1.2) | 0 (0.0) |  |  | 8 (1.1) | 3 (1.7) | 0 (0.0) | 5 (1.2) | 0 (0.0) |  |
| 2013-2014 | 41 (5.8) | 4 (2.2) | 0 (0.0) | 32 (7.9) | 5 (6.3) |  |  | 41 (5.8) | 4 (2.2) | 0 (0.0) | 32 (7.9) | 5 (6.3) |  |
| 2015-2016 | 105 (14.8) | 17 (9.6) | 5 (10.2) | 58 (14.3) | 25 (31.6) |  |  | 105 (14.8) | 17 (9.6) | 5 (10.2) | 58 (14.3) | 25 (31.6) |  |
| 2017-2018 | 162 (22.8) | 30 (16.9) | 16 (32.7) | 97 (24.0) | 19 (24.1) |  |  | 162 (22.8) | 30 (16.9) | 16 (32.7) | 97 (24.0) | 19 (24.1) |  |
| 2019-2020 | 276 (38.8) | 83 (46.6) | 21 (42.9) | 148 (36.5) | 24 (30.4) |  |  | 276 (38.8) | 83 (46.6) | 21 (42.9) | 148 (36.5) | 24 (30.4) |  |
| 2021 | 111 (15.6) | 41 (23.0) | 6 (12.2) | 58 (14.3) | 6 (7.6) |  |  | 111 (15.6) | 41 (23.0) | 6 (12.2) | 58 (14.3) | 6 (7.6) |  |
| Menopausal status at baseline (%) |  |  |  |  |  |  |  |  |  |  |  |  |  |
| Premenopausal | 313 (44.0) | 76 (42.7) | 22 (44.9) | 175 (43.2) | 40 (50.6) | 0.844 |  | 313 (44.0) | 76 (42.7) | 22 (44.9) | 175 (43.2) | 40 (50.6) | 0.844 |
| Postmenopausal | 355 (49.9) | 90 (50.6) | 23 (46.9) | 208 (51.4) | 34 (43.0) |  |  | 355 (49.9) | 90 (50.6) | 23 (46.9) | 208 (51.4) | 34 (43.0) |  |
| Missing data | 43 (6.0) | 12 (6.7) | 4 (8.2) | 22 (5.4) | 5 (6.3) |  |  | 43 (6.0) | 12 (6.7) | 4 (8.2) | 22 (5.4) | 5 (6.3) |  |
| **Clinical T stage (cT; %)** |  |  |  |  |  |  |  |  |  |  |  |  |  |
| T1 | 127 (17.9) | 27 (15.2) | 8 (16.3) | 80 (19.8) | 12 (15.2) | 0.507 |  | 127 (17.9) | 27 (15.2) | 8 (16.3) | 80 (19.8) | 12 (15.2) | 0.507 |
| T2 | 584 (82.1) | 151 (84.8) | 41 (83.7) | 325 (80.2) | 67 (84.8) |  |  | 584 (82.1) | 151 (84.8) | 41 (83.7) | 325 (80.2) | 67 (84.8) |  |
| Clinical stage (cTNM)(%) |  |  |  |  |  |  |  |  |  |  |  |  |  |
| IA | 127 (17.9) | 27 (15.2) | 8 (16.3) | 80 (19.8) | 12 (15.2) | 0.507 |  | 127 (17.9) | 27 (15.2) | 8 (16.3) | 80 (19.8) | 12 (15.2) | 0.507 |
| IIA | 584 (82.1) | 151 (84.8) | 41 (83.7) | 325 (80.2) | 67 (84.8) |  |  | 584 (82.1) | 151 (84.8) | 41 (83.7) | 325 (80.2) | 67 (84.8) |  |
| Pathological subtype (%) |  |  |  |  |  |  |  |  |  |  |  |  |  |
| TNBC | 684 (96.2) | 172 (96.6) | 46 (93.9) | 391 (96.5) | 75 (94.9) | 0.055 |  | 684 (96.2) | 172 (96.6) | 46 (93.9) | 391 (96.5) | 75 (94.9) | 0.055 |
| Luminal A | 15 (2.1) | 1 (0.6) | 1 (2.0) | 10 (2.5) | 3 (3.8) |  |  | 15 (2.1) | 1 (0.6) | 1 (2.0) | 10 (2.5) | 3 (3.8) |  |
| Luminal B/HER2- | 6 (0.8) | 1 (0.6) | 2 (4.1) | 3 (0.7) | 0 (0.0) |  |  | 6 (0.8) | 1 (0.6) | 2 (4.1) | 3 (0.7) | 0 (0.0) |  |
| Non-luminal HER2+ | 5 (0.7) | 4 (2.2) | 0 (0.0) | 0 (0.0) | 1 (1.3) |  |  | 5 (0.7) | 4 (2.2) | 0 (0.0) | 0 (0.0) | 1 (1.3) |  |
| Unspecified (ER-,PR+,HER2-) | 1 (0.1) | 0 (0.0) | 0 (0.0) | 1 (0.2) | 0 (0.0) |  |  | 1 (0.1) | 0 (0.0) | 0 (0.0) | 1 (0.2) | 0 (0.0) |  |
| Pathological T stage (ypT; %) |  |  |  |  |  |  |  |  |  |  |  |  |  |
| T0 | 19 (2.7) | 1 (0.6) | 0 (0.0) | 13 (3.2) | 5 (6.3) | <0.001 |  | 19 (2.7) | 1 (0.6) | 0 (0.0) | 13 (3.2) | 5 (6.3) | <0.001 |
| T1 | 303 (42.6) | 121 (68.0) | 28 (57.1) | 127 (31.4) | 27 (34.2) |  |  | 303 (42.6) | 121 (68.0) | 28 (57.1) | 127 (31.4) | 27 (34.2) |  |
| T2 | 100 (14.1) | 44 (24.7) | 14 (28.6) | 35 (8.6) | 7 (8.9) |  |  | 100 (14.1) | 44 (24.7) | 14 (28.6) | 35 (8.6) | 7 (8.9) |  |
| T3 | 15 (2.1) | 7 (3.9) | 0 (0.0) | 7 (1.7) | 1 (1.3) |  |  | 15 (2.1) | 7 (3.9) | 0 (0.0) | 7 (1.7) | 1 (1.3) |  |
| Missing data | 274 (38.5) | 5 (2.8) | 7 (14.3) | 223 (55.1) | 39 (49.4) |  |  | 274 (38.5) | 5 (2.8) | 7 (14.3) | 223 (55.1) | 39 (49.4) |  |
| Pathological N stage (ypN; %) |  |  |  |  |  |  |  |  |  |  |  |  |  |
| N0 | 557 (78.3) | 132 (74.2) | 43 (87.8) | 318 (78.5) | 64 (81.0) | <0.001 |  | 557 (78.3) | 132 (74.2) | 43 (87.8) | 318 (78.5) | 64 (81.0) | <0.001 |
| N1 | 99 (13.9) | 33 (18.5) | 2 (4.1) | 60 (14.8) | 4 (5.1) |  |  | 99 (13.9) | 33 (18.5) | 2 (4.1) | 60 (14.8) | 4 (5.1) |  |
| N2 | 19 (2.7) | 7 (3.9) | 2 (4.1) | 10 (2.5) | 0 (0.0) |  |  | 19 (2.7) | 7 (3.9) | 2 (4.1) | 10 (2.5) | 0 (0.0) |  |
| N3 | 2 (0.3) | 0 (0.0) | 1 (2.0) | 0 (0.0) | 1 (1.3) |  |  | 2 (0.3) | 0 (0.0) | 1 (2.0) | 0 (0.0) | 1 (1.3) |  |
| NX | 34 (4.8) | 6 (3.4) | 1 (2.0) | 17 (4.2) | 10 (12.7) |  |  | 34 (4.8) | 6 (3.4) | 1 (2.0) | 17 (4.2) | 10 (12.7) |  |
| Pathological stage (ypTNM)(%) |  |  |  |  |  |  |  |  |  |  |  |  |  |
| IA | 241 (33.9) | 98 (55.1) | 27 (55.1) | 95 (23.5) | 21 (26.6) | <0.001 |  | 241 (33.9) | 98 (55.1) | 27 (55.1) | 95 (23.5) | 21 (26.6) | <0.001 |
| IIA | 112 (15.8) | 44 (24.7) | 12 (24.5) | 45 (11.1) | 11 (13.9) |  |  | 112 (15.8) | 44 (24.7) | 12 (24.5) | 45 (11.1) | 11 (13.9) |  |
| IIB | 28 (3.9) | 18 (10.1) | 0 (0.0) | 10 (2.5) | 0 (0.0) |  |  | 28 (3.9) | 18 (10.1) | 0 (0.0) | 10 (2.5) | 0 (0.0) |  |
| IIIA | 22 (3.1) | 8 (4.5) | 2 (4.1) | 12 (3.0) | 0 (0.0) |  |  | 22 (3.1) | 8 (4.5) | 2 (4.1) | 12 (3.0) | 0 (0.0) |  |
| IIIC | 1 (0.1) | 0 (0.0) | 0 (0.0) | 0 (0.0) | 1 (1.3) |  |  | 1 (0.1) | 0 (0.0) | 0 (0.0) | 0 (0.0) | 1 (1.3) |  |
| Unspecified | 307 (43.2) | 10 (5.6) | 8 (16.3) | 243 (60.0) | 46 (58.2) |  |  | 307 (43.2) | 10 (5.6) | 8 (16.3) | 243 (60.0) | 46 (58.2) |  |
| NHG (%) |  |  |  |  |  |  |  |  |  |  |  |  |  |
| Grade1 | 10 (1.4) | 4 (2.2) | 0 (0.0) | 5 (1.2) | 1 (1.3) | <0.001 |  | 10 (1.4) | 4 (2.2) | 0 (0.0) | 5 (1.2) | 1 (1.3) | <0.001 |
| Grade2 | 136 (19.1) | 63 (35.4) | 12 (24.5) | 54 (13.3) | 7 (8.9) |  |  | 136 (19.1) | 63 (35.4) | 12 (24.5) | 54 (13.3) | 7 (8.9) |  |
| Grade3 | 204 (28.7) | 82 (46.1) | 28 (57.1) | 79 (19.5) | 15 (19.0) |  |  | 204 (28.7) | 82 (46.1) | 28 (57.1) | 79 (19.5) | 15 (19.0) |  |
| Missing data | 361 (50.8) | 29 (16.3) | 9 (18.4) | 267 (65.9) | 56 (70.9) |  |  | 361 (50.8) | 29 (16.3) | 9 (18.4) | 267 (65.9) | 56 (70.9) |  |
| Survival status (%) |  |  |  |  |  |  |  |  |  |  |  |  |  |
| Alive | 643 (90.4) | 167 (93.8) | 39 (79.6) | 367 (90.6) | 70 (88.6) | 0.121 |  | 643 (90.4) | 167 (93.8) | 39 (79.6) | 367 (90.6) | 70 (88.6) | 0.121 |
| Death by BC | 50 (7.0) | 7 (3.9) | 7 (14.3) | 29 (7.2) | 7 (8.9) |  |  | 50 (7.0) | 7 (3.9) | 7 (14.3) | 29 (7.2) | 7 (8.9) |  |
| Death by other causes | 18 (2.5) | 4 (2.2) | 3 (6.1) | 9 (2.2) | 2 (2.5) |  |  | 18 (2.5) | 4 (2.2) | 3 (6.1) | 9 (2.2) | 2 (2.5) |  |
| **Charlson comorbidity index, weighted, grouped (%)** |  |  |  |  |  |  |  |  |  |  |  |  |  |
| CCIw=0 | 583 (82.0) | 147 (82.6) | 37 (75.5) | 336 (83.0) | 63 (79.7) | 0.802 |  | 583 (82.0) | 147 (82.6) | 37 (75.5) | 336 (83.0) | 63 (79.7) | 0.802 |
| CCIw=1-3 | 126 (17.7) | 30 (16.9) | 12 (24.5) | 68 (16.8) | 16 (20.3) |  |  | 126 (17.7) | 30 (16.9) | 12 (24.5) | 68 (16.8) | 16 (20.3) |  |
| CCIw=4-10 | 2 (0.3) | 1 (0.6) | 0 (0.0) | 1 (0.2) | 0 (0.0) |  |  | 2 (0.3) | 1 (0.6) | 0 (0.0) | 1 (0.2) | 0 (0.0) |  |
| Surgery (%) |  |  |  |  |  |  |  |  |  |  |  |  |  |
| Mastectomy | 232 (32.6) | 41 (23.0) | 35 (71.4) | 87 (21.5) | 69 (87.3) | <0.001 |  | 232 (32.6) | 41 (23.0) | 35 (71.4) | 87 (21.5) | 69 (87.3) | <0.001 |
| Breast-conserving surgery | 460 (64.7) | 136 (76.4) | 6 (12.2) | 314 (77.5) | 4 (5.1) |  |  | 460 (64.7) | 136 (76.4) | 6 (12.2) | 314 (77.5) | 4 (5.1) |  |
| Subcutaneous mastectomy | 17 (2.4) | 1 (0.6) | 8 (16.3) | 3 (0.7) | 5 (6.3) |  |  | 17 (2.4) | 1 (0.6) | 8 (16.3) | 3 (0.7) | 5 (6.3) |  |
| Only axilla surgery | 0 (0.0) | 0 (0.0) | 0 (0.0) | 0 (0.0) | 0 (0.0) |  |  | 0 (0.0) | 0 (0.0) | 0 (0.0) | 0 (0.0) | 0 (0.0) |  |
| Missing data | 2 (0.3) | 0 (0.0) | 0 (0.0) | 1 (0.2) | 1 (1.3) |  |  | 2 (0.3) | 0 (0.0) | 0 (0.0) | 1 (0.2) | 1 (1.3) |  |
| Axillary surgery (%) |  |  |  |  |  |  |  |  |  |  |  |  |  |
| SN | 500 (70.3) | 126 (70.8) | 37 (75.5) | 278 (68.6) | 59 (74.7) | 0.83 |  | 500 (70.3) | 126 (70.8) | 37 (75.5) | 278 (68.6) | 59 (74.7) | 0.83 |
| ALND | 93 (13.1) | 20 (11.2) | 6 (12.2) | 60 (14.8) | 7 (8.9) |  |  | 93 (13.1) | 20 (11.2) | 6 (12.2) | 60 (14.8) | 7 (8.9) |  |
| SN and ALND | 102 (14.3) | 28 (15.7) | 5 (10.2) | 59 (14.6) | 10 (12.7) |  |  | 102 (14.3) | 28 (15.7) | 5 (10.2) | 59 (14.6) | 10 (12.7) |  |
| Sampling | 0 (0.0) | 0 (0.0) | 0 (0.0) | 0 (0.0) | 0 (0.0) |  |  | 0 (0.0) | 0 (0.0) | 0 (0.0) | 0 (0.0) | 0 (0.0) |  |
| Missing data | 16 (2.3) | 4 (2.2) | 1 (2.0) | 8 (2.0) | 3 (3.8) |  |  | 16 (2.3) | 4 (2.2) | 1 (2.0) | 8 (2.0) | 3 (3.8) |  |
| Neoadjuvant chemotherapy (%) |  |  |  |  |  |  |  |  |  |  |  |  |  |
| Yes | 711 (100.0) | 178 (100.0) | 49 (100.0) | 405 (100.0) | 79 (100.0) | NA |  | 711 (100.0) | 178 (100.0) | 49 (100.0) | 405 (100.0) | 79 (100.0) | NA |
| No | 0 (0.0) | 0 (0.0) | 0 (0.0) | 0 (0.0) | 0 (0.0) |  |  | 0 (0.0) | 0 (0.0) | 0 (0.0) | 0 (0.0) | 0 (0.0) |  |
| Completed NACT according to plan (%) |  |  |  |  |  |  |  |  |  |  |  |  |  |
| Yes | 477 (67.1) | 101 (56.7) | 30 (61.2) | 290 (71.6) | 56 (70.9) | 0.015 |  | 477 (67.1) | 101 (56.7) | 30 (61.2) | 290 (71.6) | 56 (70.9) | 0.015 |
| No | 231 (32.5) | 75 (42.1) | 19 (38.8) | 114 (28.1) | 23 (29.1) |  |  | 231 (32.5) | 75 (42.1) | 19 (38.8) | 114 (28.1) | 23 (29.1) |  |
| Missing data | 3 (0.4) | 2 (1.1) | 0 (0.0) | 1 (0.2) | 0 (0.0) |  |  | 0 (0.0) | 2 (1.1) | 0 (0.0) | 1 (0.2) | 0 (0.0) |  |
| Reason for discontinuation of NACT (%) |  |  |  |  |  |  |  |  |  |  |  |  |  |
| Side effects | 177 (24.9) | 57 (32.0) | 11 (22.4) | 95 (23.5) | 14 (17.7) | 0.003 |  | 177 (24.9) | 57 (32.0) | 11 (22.4) | 95 (23.5) | 14 (17.7) | 0.003 |
| Other | 53 (7.5) | 17 (9.6) | 8 (16.3) | 19 (4.7) | 9 (11.4) |  |  | 53 (7.5) | 17 (9.6) | 8 (16.3) | 19 (4.7) | 9 (11.4) |  |
| Missing data | 481 (67.6) | 104 (58.5) | 30 (61.2) | 291 (71.9) | 56 (70.9) |  |  | 481 (67.6) | 104 (58.5) | 30 (61.2) | 291 (71.9) | 56 (70.9) |  |
| NACT administered (%) |  |  |  |  |  |  |  |  |  |  |  |  |  |
| Anthracycline-based | 67 (9.4) | 13 (7.3) | 6 (12.2) | 41 (10.1) | 7 (8.9) | 0.782 |  | 67 (9.4) | 13 (7.3) | 6 (12.2) | 41 (10.1) | 7 (8.9) | 0.782 |
| Anthracycline+taxane | 605 (85.1) | 157 (88.2) | 39 (79.6) | 342 (84.4) | 67 (84.8) |  |  | 605 (85.1) | 157 (88.2) | 39 (79.6) | 342 (84.4) | 67 (84.8) |  |
| Taxane-based | 34 (4.8) | 6 (3.4) | 4 (8.2) | 19 (4.7) | 5 (6.3) |  |  | 34 (4.8) | 6 (3.4) | 4 (8.2) | 19 (4.7) | 5 (6.3) |  |
| Unknown | 5 (0.7) | 2 (1.1) | 0 (0.0) | 3 (0.7) | 0 (0.0) |  |  | 5 (0.7) | 2 (1.1) | 0 (0.0) | 3 (0.7) | 0 (0.0) |  |
| None | 0 (0.0) | 0 (0.0) | 0 (0.0) | 0 (0.0) | 0 (0.0) |  |  | 0 (0.0) | 0 (0.0) | 0 (0.0) | 0 (0.0) | 0 (0.0) |  |
| Adjuvant chemotherapy (%) |  |  |  |  |  |  |  |  |  |  |  |  |  |
| Yes | 227 (31.9) | 178 (100.0) | 49 (100.0) | 0 (0.0) | 0 (0.0) | <0.001 |  | 227 (31.9) | 178 (100.0) | 49 (100.0) | 0 (0.0) | 0 (0.0) | <0.001 |
| No | 484 (68.1) | 0 (0.0) | 0 (0.0) | 405 (100.0) | 79 (100.0) |  |  | 484 (68.1) | 0 (0.0) | 0 (0.0) | 405 (100.0) | 79 (100.0) |  |
| Completed ACT according to plan (%) |  |  |  |  |  |  |  |  |  |  |  |  |  |
| Yes | 143 (20.1) | 115 (64.6) | 28 (57.1) | 0 (0.0) | 0 (0.0) | <0.001 |  | 143 (20.1) | 115 (64.6) | 28 (57.1) | 0 (0.0) | 0 (0.0) | <0.001 |
| No | 81 (11.4) | 60 (33.7) | 21 (42.9) | 0 (0.0) | 0 (0.0) |  |  | 81 (11.4) | 60 (33.7) | 21 (42.9) | 0 (0.0) | 0 (0.0) |  |
| Missing data | 487 (68.5) | 3 (1.7) | 0 (0.0) | 405 (100.0) | 79 (100.0) |  |  | 487 (68.5) | 3 (1.7) | 0 (0.0) | 405 (100.0) | 79 (100.0) |  |
| Reason for discontinuation of ACT (%) |  |  |  |  |  |  |  |  |  |  |  |  |  |
| Side effects | 62 (8.7) | 50 (28.1) | 12 (24.5) | 0 (0.0) | 0 (0.0) | <0.001 |  | 62 (8.7) | 50 (28.1) | 12 (24.5) | 0 (0.0) | 0 (0.0) | <0.001 |
| Other | 18 (2.5) | 9 (5.1) | 9 (18.4) | 0 (0.0) | 0 (0.0) |  |  | 18 (2.5) | 9 (5.1) | 9 (18.4) | 0 (0.0) | 0 (0.0) |  |
| Missing data | 631 (88.7) | 119 (66.9) | 28 (57.1) | 405 (100.0) | 79 (100.0) |  |  | 631 (88.7) | 119 (66.9) | 28 (57.1) | 405 (100.0) | 79 (100.0) |  |
| ACT administered (%) |  |  |  |  |  |  |  |  |  |  |  |  |  |
| Anthracycline-based | 9 (1.3) | 7 (3.9) | 2 (4.1) | 0 (0.0) | 0 (0.0) | <0.001 |  | 9 (1.3) | 7 (3.9) | 2 (4.1) | 0 (0.0) | 0 (0.0) | <0.001 |
| Anthracycline+taxane | 19 (2.7) | 13 (7.3) | 6 (12.2) | 0 (0.0) | 0 (0.0) |  |  | 19 (2.7) | 13 (7.3) | 6 (12.2) | 0 (0.0) | 0 (0.0) |  |
| Taxane-based | 12 (1.7) | 9 (5.1) | 3 (6.1) | 0 (0.0) | 0 (0.0) |  |  | 12 (1.7) | 9 (5.1) | 3 (6.1) | 0 (0.0) | 0 (0.0) |  |
| Other | 187 (26.3) | 149 (83.7) | 38 (77.6) | 0 (0.0) | 0 (0.0) |  |  | 187 (26.3) | 149 (83.7) | 38 (77.6) | 0 (0.0) | 0 (0.0) |  |
| None | 484 (68.1) | 0 (0.0) | 0 (0.0) | 405 (100.0) | 79 (100.0) |  |  | 484 (68.1) | 0 (0.0) | 0 (0.0) | 405 (100.0) | 79 (100.0) |  |
| Adjuvant radiotherapy (%) |  |  |  |  |  |  |  |  |  |  |  |  |  |
| Yes | 583 (82.0) | 178 (100.0) | 0 (0.0) | 405 (100.0) | 0 (0.0) | <0.001 |  | 583 (82.0) | 178 (100.0) | 0 (0.0) | 405 (100.0) | 0 (0.0) | <0.001 |
| No | 128 (18.0) | 0 (0.0) | 49 (100.0) | 0 (0.0) | 79 (100.0) |  |  | 128 (18.0) | 0 (0.0) | 49 (100.0) | 0 (0.0) | 79 (100.0) |  |
| Locoregional treatment (%) |  |  |  |  |  |  |  |  |  |  |  |  |  |
| BCS + ART | 450 (63.3) | 136 (76.4) | 0 (0.0) | 314 (77.5) | 0 (0.0) | <0.001 |  | 450 (63.3) | 136 (76.4) | 0 (0.0) | 314 (77.5) | 0 (0.0) | <0.001 |
| Mastectomy only | 104 (14.6) | 0 (0.0) | 35 (71.4) | 0 (0.0) | 69 (87.3) |  |  | 104 (14.6) | 0 (0.0) | 35 (71.4) | 0 (0.0) | 69 (87.3) |  |
| Masectomy + ART | 128 (18.0) | 41 (23.0) | 0 (0.0) | 87 (21.5) | 0 (0.0) |  |  | 128 (18.0) | 41 (23.0) | 0 (0.0) | 87 (21.5) | 0 (0.0) |  |
| Other | 29 (4.1) | 1 (0.6) | 14 (28.6) | 4 (1.0) | 10 (12.7) |  |  | 29 (4.1) | 1 (0.6) | 14 (28.6) | 4 (1.0) | 10 (12.7) |  |
| P-values were calculated using Chi-square test for categorical variables (with continuity correction) and ANOVA for continuous variables. Variables in bold were included in the propensity score matching. Abbreviations: ACT = Adjuvant chemotherapy; ALND = Axillary lymph node dissection; ANOVA = Analysis of variance; ART = Adjuvant radiotherapy; AT = Adjuvant therapy; BC = Breast cancer; BCS = Breast-conserving surgery; CCIw = Charlson Comorbidity Index, weighted; ER = Estrogen receptor; HER2 = Human epidermal growth factor receptor2; IQR = Interquartile range; NA = Not available; NACT = Neoadjuvant chemotherapy; NaN = Not a number; NHG = Nottingham histologic grade; NX = Nodal status unknown; PR = Progesterone receptor; PSM = Propensity score matching; SN = Sentinel node; TNBC = Triple-negative breast cancer. | | | | | | | | | | | | | |
